# Distinct Roles of IL‐4, IL‐13, and IL‐22 in Human Skin Barrier Dysfunction and Atopic Dermatitis

**DOI:** 10.1111/all.70060

**Published:** 2025-09-23

**Authors:** Paolo D'Avino, Juno Kim, Manru Li, Philipp Gessner, Patrick Westermann, Yagız Pat, Carina Beha, Claudia Traidl‐Hoffmann, Jeremy Bost, Nicolas Gaudenzio, Christoph B. Messner, Cezmi A. Akdis, Yasutaka Mitamura

**Affiliations:** ^1^ Swiss Institute of Allergy and Asthma Research (SIAF) University of Zurich Davos Switzerland; ^2^ CK CARE – Christine Kühne Center for Allergy Research and Education Davos Switzerland; ^3^ Department of Environmental Medicine, Faculty of Medicine University of Augsburg Augsburg Germany; ^4^ Institute of Environmental Medicine Helmholtz Zentrum München Augsburg Germany; ^5^ SciBase AB Sundbyberg Sweden; ^6^ Toulouse Institute for Infectious and Inflammatory Disease (Infinity), INSERM UMR1291, CNRS UMR5051 University Toulouse III Toulouse France; ^7^ Genoskin SAS Toulouse France; ^8^ Department of Dermatology, Graduate School of Medical Sciences Kyushu University Fukuoka Japan

**Keywords:** atopic dermatitis, dupilumab (anti‐IL4Rα antibody), electrical impedance spectroscopy, ex vivo human skin, inflammation, interleukin‐13, interleukin‐22, interleukin‐4, skin barrier

## Abstract

**Background:**

Atopic dermatitis (AD) is a chronic type‐2 inflammatory skin disease characterized by eczema and epithelial barrier dysfunction. Along with the type‐2 cytokines IL‐4 and IL‐13, IL‐22 contributes to AD pathogenesis. To date, most skin studies rely on reconstructed keratinocytes, which do not represent the real skin response.

**Objective:**

Here, we report the distinct effects of IL‐4, IL‐13, and IL‐22 on bio‐stabilized human skin with intact barriers and immune cells.

**Methods:**

Spatial transcriptomics on AD‐lesions and non‐lesional skin was performed. Ex vivo skin barrier integrity was evaluated using electrical impedance spectroscopy (EIS), RNA‐sequencing, and untargeted proteomics, complemented by analyses of skin biopsies from dupilumab‐treated AD patients.

**Results:**

Spatial transcriptomics demonstrated that AD lesions showed reduced expression of key barrier genes, including CLDN1, FLG, and FLG2. IL‐4, IL‐13, and IL‐22 disrupted the skin barrier in the ex vivo human skin. Combining type‐2 cytokines and IL‐22 alone downregulated genes critical for barrier function and keratinization. In addition, IL‐4 and IL‐13 downregulated antimicrobial peptides, while IL‐22 upregulated them. Interestingly, IL‐4 and IL‐13 reduced IL‐22Rα1, and IL‐22 upregulated IL‐4Rα, suggesting immune cross‐regulation. Proteomic analysis confirmed that all three cytokines (IL‐4, IL‐13, and IL‐22) reduced the expression of key skin barrier proteins, particularly filaggrin and claudin‐1. Dupilumab treatment of AD patients for 3 months restored IL‐4/IL‐13‐dysregulated genes, whereas it had limited effect on IL22‐associated pathways.

**Conclusion:**

This comprehensive study provides insights into the distinct immune profiles following IL‐4, IL‐13, and IL‐22 stimulation on human skin, highlighting their complex interplay in disrupting skin barrier function and modulating innate immune responses.

## Graphical abstract

This study reports the distinct effects of IL‐4, IL‐13, and IL‐22 on bio‐stabilized human skin with intact barriers and immune cells. IL‐4, IL‐13, and IL‐22 upregulate inflammatory mediators, disrupt skin barrier integrity, and reciprocally modulate IL‐4Rα and IL‐22Rα1 receptors. Both type 2 cytokine‐ and IL‐22–related genes show limited response to anti‐IL‐4Rα antibody (dupilumab), revealing complex immune‐barrier interactions in atopic dermatitis. ADAM8, A disintegrin and metalloproteinase domain‐containing protein 8; ARG1, Arginase 1; BMP7, Bone morphogenetic Protein 7; CCL, Chemokine Ligand; CTSC, Cathepsin C; FLG, Filaggrin; HAS3, hyaluronan synthase 3; HRH1, Histamine H1 receptor; IL, interleukin; KRT, Keratin; LCE1E, Late cornified envelope 1E; NREP, Neuronal regeneration related protein; NRG2, Neuregulin 2; PRECSIT, p53 Regulated carcinoma associated STAT3 activating long intergenic non‐protein coding transcript; USH1G, Usher syndrome type 1G.

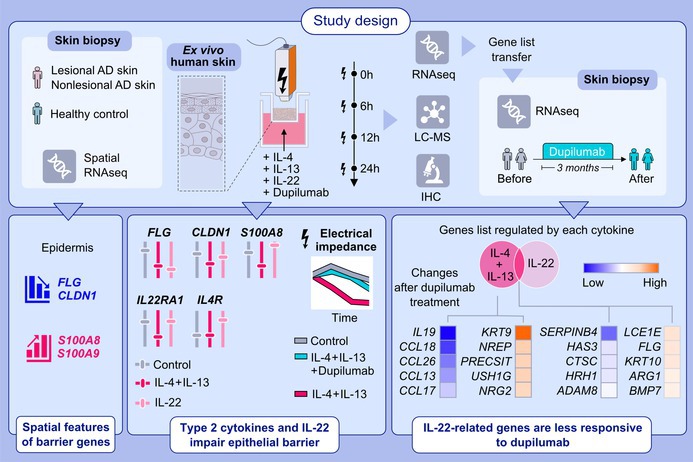

**Abbreviations**
AD: atopic dermatitis
CCL: chemokine C‐C motif ligand
CLDN: claudin
CXCL: C‐X‐C motif chemokine ligand
EASI: Eczema Area and Severity Index
EIS: electrical impedance spectroscopy
FLG: Filaggrin
GO: gene ontology (geneontology.org)
ɣc: common ɣ chain
HC: healthy control
IHC: immunohistochemistry
IL4Rα: IL‐4 receptor α‐chain
KLKs: Kallikrein‐related peptidases
LS: lesional skin
MS: mass spectrometry
NL: non‐lesional skin
PCA: principal component analysis
RNA‐Seq: RNA sequencing
scRNA‐Seq: single‐cell RNA sequencing
STAT: signal transducer and activator of transcription
TSLP: thymic stromal lymphopoietin

## 1 Introduction

Atopic dermatitis (AD) is a common chronic skin inflammatory disorder affecting 3%–5% of adults and 20% of children worldwide [1, 2, 3]. The pathogenesis of AD involves immune dysregulation, neuroinflammation, and epithelial barrier dysfunction. Immunologically, AD is primarily driven by Th2 (type‐2) and Th22 types of inflammation with varying contributions of Th1 and Th17 responses [4, 5, 6]. Blockade of type‐2 inflammation, such as with an IL‐4 receptor α‐chain blocking monoclonal antibody (mAb, e.g., dupilumab), shows significant efficacy in adults and children with moderate to severe AD. Treatment with dupilumab reduces the eczema area and severity index by approximately 75% (EASI75) in 64% of adults and 70% of children at week 16 [7, 8]. Moreover, 52%–59% of adult AD patients treated with lebrikizumab, an anti‐IL‐13 mAb, achieve EASI75 [9]. Even based on EASI50, approximately 20% of AD patients are non‐responders who show limited clinical improvement [10]. However, the presence of non‐responder AD patients suggests that other biological pathways beyond the Th2 axis might play a role. IL‐22 is another important cytokine in the pathogenesis of AD produced by Th22 cells and Th17 cells [11]. Clinical improvement was observed in AD patients with a high IL‐22 baseline treated with the IL‐22 blocking mAb, fezakinumab [12]. Understanding the roles of the key cytokines in AD pathogenesis, specifically IL‐4, IL‐13, and IL‐22, is essential for personalized treatment and identifying therapy nonresponsive patients and potential therapeutic targets.

We, and others, have reported characteristic changes in AD lesional skin [13, 14, 15, 16, 17]. The stratified keratinocytes are arranged to form the primary physical barrier against pathogens, allergens, and irritants, together with structural proteins, such as tight junctions (TJs) (e.g., claudin‐1; CLDN1 and claudin‐4; CLDN4), desmosomes (e.g., DSG1 and DSG3), adherence junctions (AJs), gap junctions, and the stratum corneum (e.g., filaggrin; FLG and loricrin; LOR). Antimicrobial peptides, such as S100A7, S100A8, and S100A9, play a central role in the innate immune defense of the skin against pathogens [18]. In addition, angiogenesis and neurogenesis are also important for regulating the skin barrier. Angiogenesis is essential for providing nutrients and oxygen to the skin, supporting its regenerative capacity [19]. Neurogenesis enhances sensory perception, enabling the detection of changes in the environment, such as temperature and exposure to toxic agents [20]. This, in turn, can trigger sensations, such as itch and pain, playing a critical role in the skin barrier.

The present study describes the distinct effects of AD‐related cytokines (IL‐4, IL‐13, and IL‐22) in the human skin, underlining their complex interaction within the heterogeneous skin cellular composition. To date, epithelial barrier research relies on reconstructed epithelia; therefore, understanding the complexity of the heterogeneous skin's immune response is still a critical challenge in advancing therapeutic strategies. Accordingly, we treated ex vivo human skin biopsies (NativeSkin, Genoskin SAS, France), exhibiting the full epidermis, dermis, and resident immune cells with IL‐4, IL‐13, IL‐4 + IL‐13, and IL‐22. We assessed the epidermal barrier integrity using electrical impedance spectroscopy (EIS), which is a validated noninvasive method to evaluate skin barrier integrity [21, 22, 23]. We identified that IL‐4, IL‐13, IL‐4 + IL‐13, and IL‐22 significantly reduced the skin barrier integrity, such as in AD lesional skin. Their characteristic changes, such as barrier impairment and pro‐inflammatory response, were demonstrated using a combination of powerful multi‐omics methods, including bulk and spatial RNA‐seq and untargeted mass spectrometry‐based proteomics. Through the analysis of our data collected from NativeSkin samples with the data from AD patients treated with dupilumab, we identified an ameliorated IL‐4 and IL‐13‐specific type‐2 response, but not a shared response with IL‐22, after 3 months of treatment. We showed that dupilumab protected the barrier function of NativeSkin samples impaired by the combination of IL‐4 and IL‐13, even with IL‐22.

## 2 Methods

### 2.1 Cytokine Treatment on Ex Vivo Human Skin: Source, Conditions, and Sampling Methods

Ex vivo human skin samples (NativeSkin) from Genoskin SAS were obtained from healthy donors who underwent abdominoplasty (Table 1). These natural human skin samples were cultured in chemically defined xeno‐free Genoskin medium at 37°C with 5% CO_2_.

**TABLE 1:** Demographic features of ex vivo skin donors in Figures 2, 3, 4, 5, 6 (donors 1–5) and in Figure 7 (donors 6–10).

Donor No	Sex/age	Skin type	Size (cm)	Weight (Kg)	BMI	Skin disease	Treatment/anatomical site
1	F/32	3	180	80	24.7	None	None/Abdomen
2	F/51	3	165	84.9	31.2	None	None/Abdomen
3	F/58	2	166	65	24	None	None/Abdomen
4	F/42	3	157	66	27	None	None/Abdomen
5	F/45	2	166	88	32	None	None/Abdomen
6	F/37	2	161	64	25	None	None/Abdomen
7	F/54	2	167	67	24	None	None/Abdomen
8	F/62	2	160	62	24	None	None/Abdomen
9	F/44	3	160	78	30	None	Azinc, Tardyferon, folic acid, calcitriol, ZymaD, calcium, colecalciferol/Abdomen
10	F/31	2	163	78	29	None	None/Abdomen

Ex vivo human skin samples were treated with IL‐4 (100 ng/mL), IL‐13 (100 ng/mL), IL‐4 + IL‐13 (100 ng/mL each), IL‐22 (100 ng/mL), dupilumab (100 μg/mL), or PBS with 0.1% BSA. The media were exchanged after 12 h of stimulation, and samples were collected after 24 h of stimulation for RNA‐seq, proteomics analyses, and immunohistochemistry (IHC) experiment. For further details, see the Data S1.

### 2.2 EIS Measurements

EIS measurements were performed using Nevisense (SciBase, Sweden) according to a previous study [23]. The electrical impedance was measured at 35 frequencies between 1 kHz and 2.5 MHz at two depths in three permutations. EIS measurements were performed at 0, 6, 12, and 24 h after cytokine treatment with triplicate readings at each time point. The EIS measurements were performed on 5 biological donors.

### 2.3 Spatial RNA Sequencing

The spatial RNA‐seq data used in this study were derived from our recent publication [14]. Skin tissues were obtained from 6 healthy control donors (aged 24–63 years, 2 males and 4 females) and 7 ad patients (aged 24–63 years, 4 males and 3 females, mean SCORAD: 44.49 ± 13.73).

The previously published spatially resolved transcriptomics (SRT) data of human skin biopsy samples was obtained from GEO under the accession number GSE197023. The analysis was conducted again in R with Seurat (version 4.4.0) [24]. The clusters were systematically annotated by comparing the top 10 marker genes from each cluster, identified by FindAllMarkers, and further refined using key markers for barrier molecules (FLG, CDSN), lower epidermis (KRT5, PMEL), and sweat glands (AQP5, DCD). For further details, see the Data S1.

### 2.4 Bulk RNA‐Seq in Ex Vivo Human Skin

Briefly, one‐eighth of 15 mm diameter ex vivo human skin was minced and digested in Trizol and processed with Precellys devices (Precellys 24 homogenizer) at 4°C to extract RNA from the tissue. The RNA was then purified using an RNAeasy Plus Micro Kit (Qiagen) following the manufacturer's guidelines. RNA library preparation and sequencing were performed in two batches, with the resulting data combined for subsequent analysis (total *n* = 5 samples). The raw reads were processed using Cutadapt (version 2.5) [25]. Processed reads were aligned to the Ensembl GRCh38 human genome assembly and annotated with the Ensembl GRCh38.109 gtf file using STAR (version 2.7.9a) [26]. Exons were counted using the featureCounts read summarization function from the Subread package (version 2.0.1) [27]. For further details, see the Data S1.

All analyses, including those performed on already published data sets, were performed in the R programming language (version 4.4.1) [28]. The dupilumab‐treated human skin RNA‐Seq data was obtained from Gene Expression Omnibus (GEO) under the accession number GSE157194 [29]. Differential gene expression analysis was performed by fitting negative binomial generalized linear models followed by quasi‐likelihood F‐tests using edgeR (version 4.2.1) [30]. In the native skin samples, the linear model accounts for the biological variation between different donors. In the dupilumab‐treated human skin data, a paired design was used in order to account for the differences in the effect on each individual patient. Enrichment analysis for gene ontology was performed in topGO (version 2.56.0) [31], using the Elim algorithm combined with Fisher's exact test. All heatmaps were generated using ComplexHeatmap (version 2.20.0) [32].

### 2.5 Sample Processing for LC–MS

Ex vivo skins were disrupted in RIPA lysis buffer (1:10, EMD Millipore Sigma‐Aldrich, MA, USA) with protease inhibitor (cOmplete tablets, Mini EDTA‐free, EASYpack, Roche, Switzerland). Afterward, ex vivo skins were homogenized using CK‐Mix beads (Bertin, France) and the Precellys 24 homogenizer. Samples were normalized to a concentration of 0.5 mg/mL with the 1X RIPA lysis buffer.

Samples were prepared according to the SP4 protocols (*n* = 3 biological donors) [33].

Briefly, protein lysates were sonicated and mixed with 100 mM tris (2‐carboxyethyl) phosphine/400 mM chloroacetamide. The proteins were precipitated by adding acetonitrile. The plate was mixed for 5 s at 400 rpm. The plate was centrifuged, and the precipitate was washed three times with 200 μL of 80% ethanol. Finally, 100 mM ABC (pH 8.0) was added to the wells; the plate was sonicated for 5 min. Then, 0.05 μg/μL Trypsin/Lys‐C was added to the wells, and the plate was incubated for 18 h at 37°C and 500 rpm. The digestion was stopped by the addition of 10% formic acid. The peptides were desalted using an Oasis PRiME HLB 96‐well μElution Plate. Samples were loaded onto the plate, the wells were washed twice with 0.1% formic acid (FA) and once with ddH_2_O, and eluted with 70% acetonitrile after a 1‐min incubation at room temperature. The samples were resuspended in 3% acetonitrile and 0.1% FA, then were sonicated for 5 min. For further details, see the Data S1.

### 2.6 LC–MS Measurements

Samples were acquired on an EASY‐nLC1200 coupled to a Thermo Orbitrap Eclipse Tribrid MS in data‐independent acquisition (DIA) mode. Buffer A consisted of 0.1% formic acid in water and buffer B 0.1% formic acid in 80% acetonitrile. A nanoLC column with an integrated emitter from CoAnn Technologies was used with the following dimensions: 75 μm ID × 25 cm L × 365 μm OD, ReproSil‐Pur120 C18 particles (1.9 μm). An Acclaim PepMap 100 C18 pre‐column was used (0.1 mm ID × 150 mm L with 5 μm particle size). Flow rate was set to 400 nL/min. For further details, see the Data S1.

### 2.7 Proteomic Data Analysis

Raw data was processed using DIA‐NN software with default settings [34]. Downstream analysis was performed with R. Differential expression analysis was performed using the limma R package, incorporating the donors into the models [35]. The linear models were fitted protein‐wise using the lmFit function within the limma package. The t‐statistics were computed using the eBayes function, allowing for an intensity trend in the prior variance. For further details, see the Data S1.

### 2.8 Immunohistochemistry Staining

Frozen ex vivo skin sections (7 μm) from 4 donors under 5 different conditions (control, IL‐4, IL‐13, IL‐4 + IL‐13, IL‐22) were fixed with 4% paraformaldehyde (Fluka, St Louis, Mo). Permeabilization and blocking were accomplished by incubating a mixture of 10% goat serum (DakoCytomation, Glostrup, Denmark), PBS containing 0.2% Triton X (Acros Organics, Geel, Belgium), and 1% BSA (Sigma‐Aldrich) in PBS. Specimens were then stained with an anti‐FLG antibody (Abcam, ab218395, mouse IgG1) and an anti‐CD45 antibody (Invitrogen, MA5‐17687, rat IgG2A). Slides were mounted with DAPI (Sigma‐Aldrich). Specimens were examined under a Zeiss LSM 780 (Carl Zeiss Microscopy GmbH, Oberkochen, Germany) in the tail mode scan, acquiring 6 pictures. 10 squares with the same sizes were applied to each picture, and the mean intensity was calculated with FIJI (ImageJ). One‐way ANOVA was applied with adjusted *p*‐values. For further details, see the Data S1.

## 3 Results

### 3.1 Spatial Transcriptomic Characteristics of Barrier Dysfunction in Human AD Skin

**FIGURE 1:**
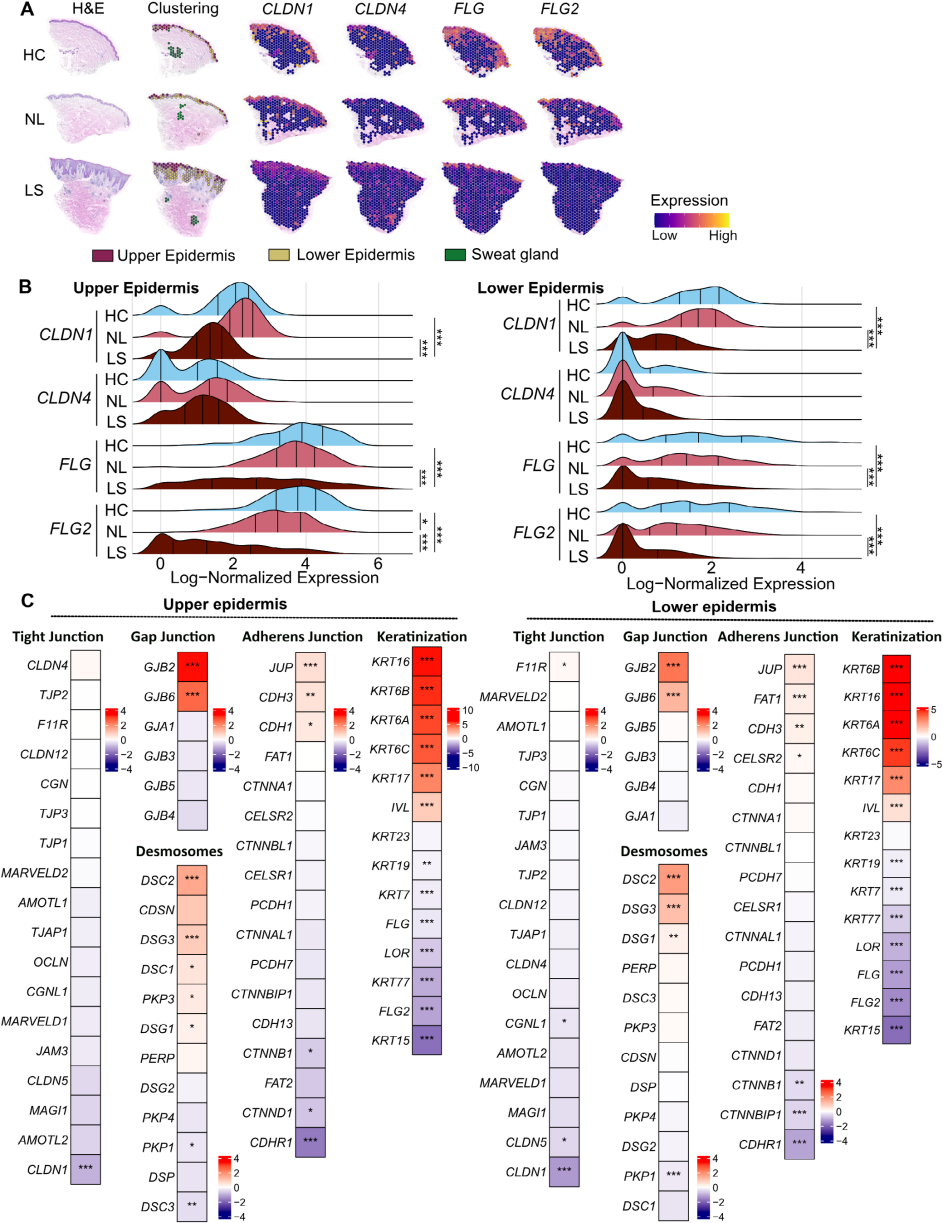
Characterization of barrier dysfunction in atopic dermatitis by spatial transcriptomics. (A) Spatial feature plots of expression of skin barrier‐related genes, claudin‐1 (*CLDN1*), (*CLDN‐4*), filaggrin (*FLG*), and *FLG‐2*. The representative images of healthy control (HC), nonlesional AD (NL), and lesional AD (LS) are shown with the clustering; the upper epidermis (red), lower epidermis (yellow), and sweat glands (green). (B) Ridgeline plots of the indicated gene expression within the upper and lower epidermis clusters *p < 0.05, **p < 0.01, ***p < 0.001, Wilcoxon rank‐sum test. (C) Heatmaps show the expression changes (average log2 fold change) of skin barrier gene families: Tight junctions, adherens junctions, gap junctions, desmosomes, adherens junctions, and keratinization, in lesional AD skin, compared to healthy controls, in both upper and lower epidermis clusters.

To explore the transcriptomic profile of the skin barrier of AD patients during lesion formation, we performed spatial transcriptomics analysis on biopsy samples from AD lesional skin (LS), AD non‐lesional skin (NL), and healthy control skin (HC) (Figure 1A and Figure S1A). The expressions of *CLDN1*, *CLDN4*, *FLG*, *FLG2*, *DSG1*, and *DSG3* were spatially located mainly in the epidermis, and the spatial expression of *CLDN1*, *FLG*, and *FLG2*, both in the upper and lower epidermis, was downregulated in LS compared to HC and NL (Figure 1B). Other CLDN family genes, such as *CLDN5*, *CLDN7*, and *CLDN8*, were localized mainly in the dermis (Figure S1A). Their expression was overlaid with endothelial cells and sweat glands in the dermis. In addition, other important TJ molecules, such as *OCLN*, *TJP1*, and *TJP2*, were not downregulated in AD lesions (Figure S1A,B). Sub‐heatmaps focusing on the barrier genes indicated that *DSC3*, *PKP1*, *LOR*, *KRT7*, *KRT15*, *KRT19*, *KRT77*, and *CDHR1* were also downregulated in the epidermis of lesional skin. Conversely, we found that many barrier‐related genes, such as *DSG1*, *DSG3*, *DSC2*, *GJB2*, and *GJB6*, are significantly upregulated in the upper and lower epidermis of lesional AD skin (Figure 1C and Figure S1A,B). The keratins, *KRT6A‐C*, *KRT16*, *KRT17*, and involucrin (*IVL*), were significantly upregulated in the lesional epidermis compared to the control epidermis (Figure 1C).

### 3.2 Type‐2 Inflammation Impairs the Epithelial Barrier in Ex Vivo Human Skin

**FIGURE 2:**
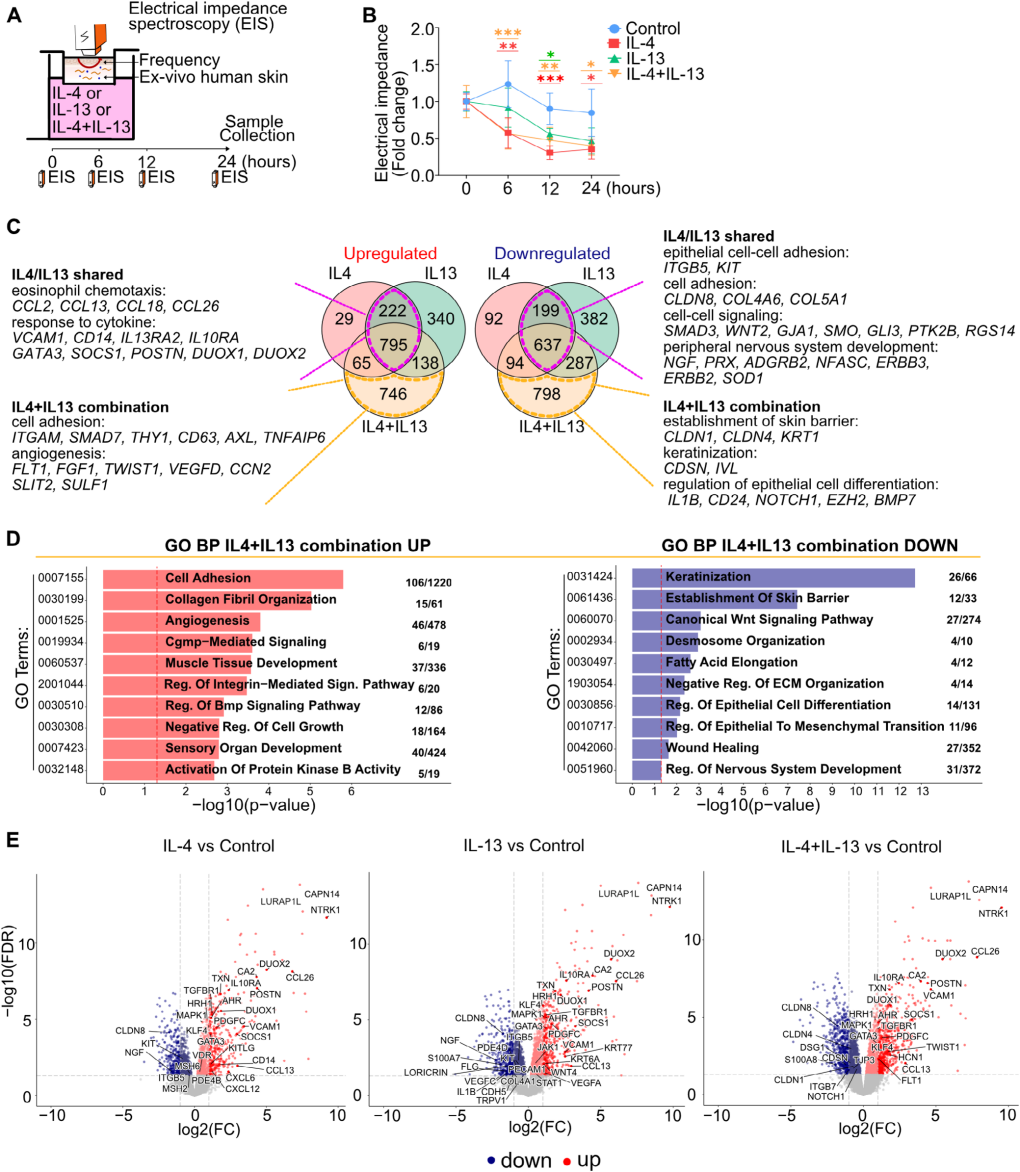
Type‐2 cytokine exposure decreases electrical impedance and downregulates the expression of skin barrier‐related genes in ex vivo human skin. (A) Ex vivo human. Skin (NativeSkin, *n* = 5 biological donors) samples were treated with IL‐4 (100 ng/mL), IL‐13 (100 ng/mL), and IL‐4 and IL‐13 (100 ng/mL each) for 24 h. (B) Electrical impedance measurements were performed on 5 donors before and after the cytokine stimulations at indicated time points (0, 6, 12, 24 h). The data were normalized with the values at 0 h in each sample. A two‐way ANOVA test was used to compare differences between each stimulation and control at each time point. Data are shown as mean ± SEM. The star colors indicate the statistical significance corresponding to the colors of the stimulations shown in the legend. *: *p* < 0.05, **: *p* < 0.01, ***: *p* < 0.001. (C) Venn diagrams illustrate the number of significantly differentially expressed genes (*n* = 5 biological donors; FDR < 0.05) in comparison to both stimulations and the control. Purple dotted line: IL‐4/IL‐13 shared, genes modulated by stimulation of IL‐4 or IL‐13 alone. Yellow dotted line: IL‐4 + IL‐13 combination, genes modulated only by combined IL‐4 and IL‐13, but not by each alone. The representative genes and pathways are indicated. (D) The representative significant Gene Ontology terms of biological processes (GO BP) associated with the differentially expressed genes shown in the comparison between IL‐4 + IL‐13 combination and the control sample. The numbers indicate the number of overlapping significantly differentially expressed genes/the number of involved genes in the corresponding process. (E) Volcano plots showing the differentially expressed genes in comparison with the control group. Genes with FDR of less than 0.05 are marked in red (Up) and blue (Down).

A type‐2 inflammation human skin model was developed to investigate the regulation of barrier‐related gene expression in AD skin. NativeSkin samples are skin biopsies from healthy living human donors that preserve the full architecture of human skin with tissue‐resident immune cells [23, 36]. NativeSkin samples are treated with IL‐4, IL‐13, and their combinations. EIS measurements were recorded before treatment and at various time points (Figure 2A). As shown in Figure 2B, after 6 h, IL‐4 and the combination of IL‐4 and IL‐13 significantly reduced the electrical impedance, and IL‐13 alone reached similar values after 12 h. These findings demonstrate the damage induced by IL‐4 and IL‐13 on the epithelial barrier integrity in ex vivo human skin.

### 3.3 The Combination of IL‐4 and IL‐13 Suppresses the Expression of Skin Barrier, Epithelial Cell Differentiation, and Keratinization Genes

To analyze the effects of IL‐4, IL‐13, and their combination on the human skin transcriptome, we isolated the RNA after 24 h of stimulation and performed RNA sequencing. Each stimulation significantly altered the transcriptome (Figure 2C and Table S1). IL‐4, IL‐13, and their combination induced the upregulation of 1111, 1495, and 1744 genes, and downregulated 1022, 1505, and 1816 genes, respectively. 1017 upregulated and 836 downregulated genes were shared in IL‐4 and IL‐13 alone stimulations. Some significantly up‐and downregulated genes were found to be the same for each of the cytokines, such as *CCL2*, *CCL13*, *CCL18*, *CCL26*, *POSTN*, *SOCS1*, *GATA3*, *IL13RA2*, *IL10RA*, *DUOX1*, *DUOX2*, *NTRK1*, *CAPN14*, and *LURAP1L*. As shown in Figure S2A, 94 upregulated and 186 downregulated genes were significantly modulated by IL‐4, but not IL‐13. On the other hand, 478 upregulated and 669 downregulated genes were significantly altered by IL‐13, but not IL‐4. Moreover, 746 upregulated and 798 downregulated genes were significantly modulated by the combination of IL‐4 and IL‐13. Through this transcriptomic analysis, we identified genes that were specifically stimulated or suppressed by IL‐4, IL‐13, and their combination. In addition, the differentially expressed genes (DEGs) from each sample were subjected to GO term enrichment analysis to identify the biological processes altered by these cytokines (Figure 2D; Figure S2B–D; Table S2).

Among the genes shared by IL‐4 and IL‐13 alone stimulation (IL‐4/IL‐13 shared), biological processes significantly enriched in upregulated genes were mainly associated with a typical type‐2 inflammatory response, such as eosinophil chemotaxis and response to cytokine, including *CCL2*, *CCL13*, *CCL18*, *CCL26*, *VCAM1*, *CD14*, *IL13RA2*, *IL10RA*, *GATA3*, *POSTN*, and *SOCS1*. On the other hand, pathways enriched in downregulated genes shared by IL‐4 and IL‐13 include epithelial cell adhesion and cell–cell signaling, including *CLDN8*, *KIT*, and *ITGB5*, as well as peripheral nervous system development, such as *NGF* and *PRX* (Figure 2C and Figure S2B). IL‐4 alone stimulation upregulated genes related to tumor necrosis factor production and leukocyte homeostasis, including *PDE4B*, *CXCL6*, *CASP3*, and *KITLG*, and leukocyte chemotaxis, including *CCL17*, *CXCL12*, *and CXCL6*. Moreover, we observed a negative regulation of Schwann cell proliferation genes, such as *SKI* and *SOX10*, somatic hypermutation of immunoglobulin genes, such as *MSH2* and *MSH6*, and epithelial structure maintenance, including *CROCC* and *TFF3* in downregulated genes (Figure S2A,C). In the IL‐13 alone stimulated samples, we observed an overexpression of genes related to polarized epithelial cell differentiation, including *RAB10*, *RHOA*, and *YAP1*; tricarboxylic acid cycle, including *FH*, *SUCLG1*, *MDH1*; mitochondrial respiratory chain complex assembly, including *NDUFA10*; and negative regulation of epithelial cell differentiation, such as *STAT1*, *DLL1*, and *VEGFA*. In contrast, downregulated genes were related to angiogenesis, including *TIE1*, *FLT1*, *FLT4*, *and VEGFC*; positive regulation of activated T‐cell proliferation, including *HMGB1* and *TNFSF9*; establishment of the endothelial barrier, including *PDE4D*, *CDH5*, *PECAM1*; and defense response to bacteria, including *IL22RA1*, *S100A8*, *S100A9*, and *TLR2* (Figure S2A,D). Samples stimulated with a combination of IL‐4 and IL‐13 exhibited a strongly enriched gene expression of pathways involved in AD pathogenesis. In contrast, there was a suppression of genes belonging to barrier‐related pathways such as keratinization, establishment of the skin barrier, desmosome organization, and regulation of epithelial cell differentiation, including *CLDN1*, *CLDN4*, *KRT1*, and *CDSN* (Figure 2C,D). Figure 2E shows the significantly differentiated genes from the NativeSkin samples stimulated with IL‐4 and IL‐13 alone, and in combination. The type‐2 cytokines were found to significantly upregulate *CAPN14*, *LURAP1L*, *DUOX1*, *DUOX2*, and *NTRK1*. Genes related to a defense response to bacteria, such as S100A7 and S100A8 were downregulated by IL‐13 alone and the IL‐4/IL‐13 combination. Overall, there are genes that are regulated by IL‐4 alone and IL‐13 alone and genes that are regulated by their overlapping effects.

### 3.4 Unique Transcriptomic Signatures of IL‐4 and IL‐13 Alone and in Combination in the Skin

**FIGURE 3:**
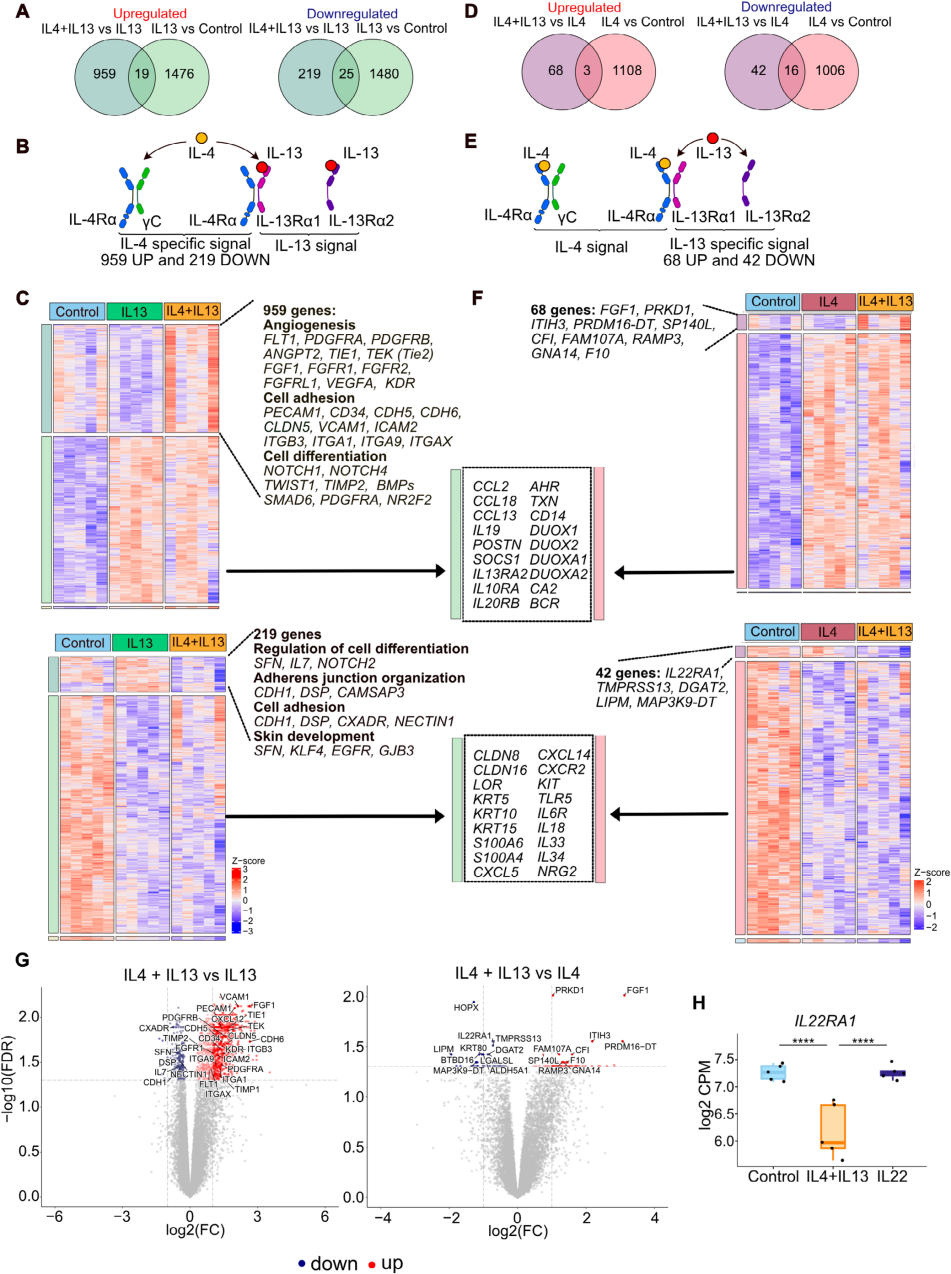
IL‐4 strongly alters gene expression under IL‐13‐induced inflammation. (A, D) Venn diagrams illustrate the differentially expressed genes (FDR < 0.05) in NativeSkin stimulated with IL‐4 and IL‐13 alone, both IL‐4/IL‐13 and the control group (*n* = 5 biological donors). (B, E) Schematic figures of the receptors for IL‐4 and IL‐13. (C, F) Heatmaps show the differentially expressed genes identified in (A, D). The representative genes in each cluster are depicted. (G) Volcano plots show the differentially expressed genes in comparison with the indicated groups. Genes with FDR of less than 0.05 are marked in red (Up) and blue (Down). (H) Gene expression of *IL22RA1* upon IL‐4 + IL‐13 and IL‐22 stimulation. **** *p* < 0.0001.

We further explored the gene expression profiles induced by IL‐4 and IL‐13 in NativeSkin samples. The combination of IL‐4 and IL‐13 was compared with IL‐4 and IL‐13 alone. The addition of IL‐4 to an IL‐13 stimulated sample significantly upregulated 959 genes and downregulated 219 genes compared to IL‐13 alone (Figure 3A–C and Table S1). These genes represent a large cluster that denotes the transcriptomic profile of the IL4Rα and common gamma chain (γc), the heterodimer of IL‐4R (Figure 3B). The upregulated cluster was found to be involved in angiogenesis, such as *TIE1*, *FLT1*, *FGF1*, and *VEGFA*; cell adhesion, such as *CLDN5*, *VCAM1*, *CDH5*, *CDH6*; and cell differentiation, such as *NOTCH1*, *NOTCH4*, *TWIST1*. Interestingly, stimulation with IL‐13 alone significantly suppressed the angiogenesis pathway, whereas an upregulation was observed in combination with IL‐4. The downregulated genes were associated with pathways involved in the regulation of cell differentiation, such as *SFN*, *IL7*, *NOTCH2*; adherens junction organization, including *CDH1*, *DSP*, *CXADR*; and skin development, such as *SFN*, *KLF4*, *EGFR*, and *GJB3*.

Interestingly, the addition of IL‐13 to IL‐4 stimulation did not have such a pronounced effect (Figure 3D–F). Many genes regulated by IL‐4 show the same trend with the regulation induced by IL‐13 and IL‐4 + IL‐13, as shown in the middle between Figure 3C,F. Only 68 genes were found to be upregulated, including *FGF1* and *PRKD1*. In contrast, 42 genes were downregulated, such as *IL22RA1* and *TMPRSS13* (Figure 3F).

Overall, these findings indicate that IL‐13 suppresses angiogenesis; however, the addition of IL‐4 restores it. This additional IL‐4 signal also affects cell adhesion and cell differentiation. The addition of IL‐13 to IL‐4 stimulation had a less pronounced effect (Figure 3G). This action may highlight the effect of IL‐13RA2, a role that has not been emphasized so far. Interestingly, the combination of IL‐4 and IL‐13 negatively regulated *IL‐22RA1*, an IL‐22 receptor, and IL‐22 upregulated the expression of *IL4R*, suggesting a cross‐regulation of signaling pathways between each other's receptors (Figure 3H and Figure S3A).

### 3.5 IL‐22 Impairs the Human Epithelial Skin Barrier and Induces Antimicrobial Peptides

**FIGURE 4:**
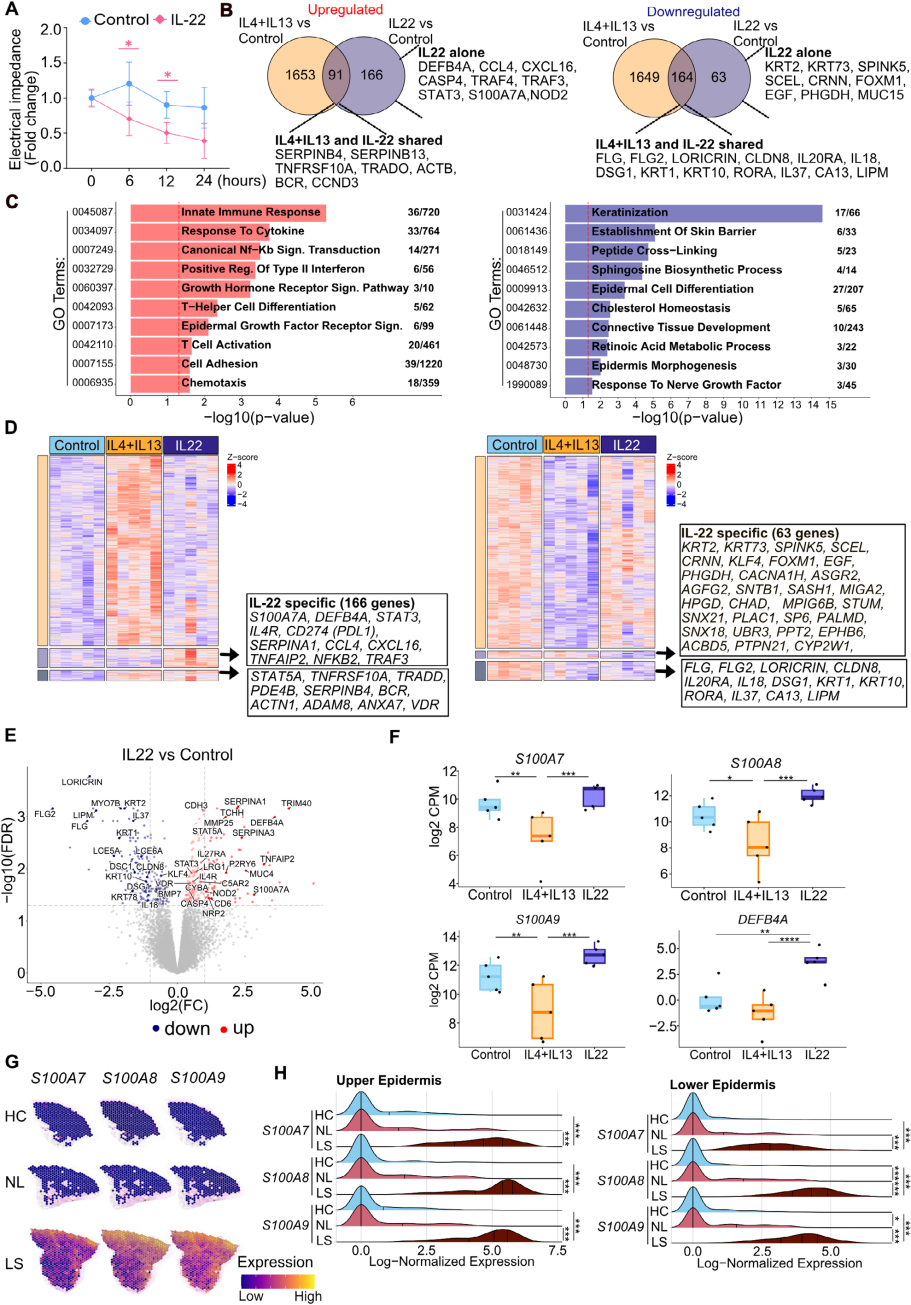
IL‐22 impairs the human skin barrier and induces antimicrobial peptides. (A) Electrical impedance measurements were performed before and after IL‐22 (100 ng/mL, *n* = 5 biological donors) treatment at the indicated time points. The data were normalized with the impedance value at 0 h for each sample. A two‐way ANOVA test was used to compare differences between each stimulation and control at each time point. Data are shown as mean ± SEM. The colored stars indicate the statistical significance. *: *p* < 0.05, **: *p* < 0.01, ***: *p* < 0.001. (B) Venn diagrams depicting the number of differentially expressed genes (FDR < 0.05) in IL‐22 alone, both IL‐4/IL‐13 and the control group. The representative genes in each intersection, IL‐4 + IL‐13 and IL‐22 shared and IL‐22 alone, are indicated. (C) The representative significant Gene Ontology terms of biological processes (GO BP) associated with the differentially expressed genes in IL‐22 compared to the control group are shown. The number indicates the number of overlapped differentially expressed genes/number of genes involved in the corresponding biological process. (D) Heatmaps show the differentially expressed genes identified in Figure 4B. The representative genes in each cluster are depicted. (E) Volcano plots show the differentially expressed genes in comparison with the indicated groups. Genes with FDR of less than 0.05 are marked in red (Up) and blue (Down). (F) Gene expression comparison between indicated antimicrobial peptides in control (CTRL), upon IL‐4 + IL‐13, and IL‐22 stimulation. (G) Spatial feature plots of expression of indicated genes in healthy control (HC), nonlesional AD (NL), and lesional AD (LS). (H) Ridge plots of the indicated gene expression within the upper and lower epidermis clusters *p ≤ 0.05, **p ≤ 0.01, ***p ≤ 0.001, ****p ≤ 0.0001, Wilcoxon rank‐sum test. (cluster identified in Figure 1A).

Since IL‐22 represents another key inflammatory cytokine in AD, its effect on the epithelial barrier was investigated in our ex vivo human skin model. Interestingly, IL‐22 also showed a significant reduction in the skin barrier integrity as measured by EIS after 6, 12, and 24 h (Figure 4A). IL‐22 induced the upregulation of 257 genes and downregulation of 227 genes, as shown in the RNA sequencing data analysis (Figure 4B; Tables S3 and S4). Upregulated genes were significantly enriched in biological processes associated with both innate and adaptive immunity, such as an innate immune response, NF‐κB signaling, T cell activation, and T‐helper cell differentiation (Figure 4C). IL‐22 significantly reduced the gene expression of key epidermal barrier‐related pathways, such as keratinization, establishment of the skin barrier, epidermal cell differentiation, and peptide cross‐linking, impacting the stability and function of genes involved in tissue repair and cell–cell adhesion. To distinguish between the specific effects of IL‐22 from type 2 cytokines, we compared the transcriptomic profile generated with IL‐22 stimulated and that with the combined IL‐4 and IL‐13 (Figure 4B,D and Table S3). Indeed, IL‐22 specifically upregulated 166 genes, such as *DEFB4A*, *S100A7A*, *CCL4*, *CXCL16*, *CASP4*, *STAT3*, and *IL4R*. Both stimulations share 91 upregulated genes, including *SERPINB4*, *SERPINB13*, *TNFRSF10A*, *ACTB*, *BCR*, and *STAT5A*. IL‐22 alone downregulated the expression of 63 genes, such as *KRT2*, *KRT73*, *SPINK5*, *EGF*, and *MUC15*. In addition, the use of both stimulations downregulated 164 genes, including *CLDN8*, *IL20RA*, *KRT10*, *LORICRIN*, *FLG*, and *FLG2*. IL‐22 reduced the gene expression of skin barrier function‐related genes but, at the same time, elevated the expression of antimicrobial peptides, including *DEFB4A*, *S100A7A*, *S100A7*, *S100A8*, and *S100A9*, particularly when compared to the effects of type 2 cytokine combinations (Figure 4E,F). *S100A7*, *S100A8*, and *S100A9* are constitutively expressed at a low level in healthy skin. They are known to be expressed in myeloid cells, endothelial cells, and epithelial cells such as keratinocytes. The spatial expression of *S100A7*, *S100A8*, and *S100A9* was upregulated in both the epidermis and dermis of AD‐lesional skin, especially on the surface of the epidermis (Figure 4G,H). Moreover, S100A13 was upregulated in the dermis of lesional AD (Figure S3B). Overall, similarly to IL‐4 and IL‐13, stimulation with IL‐22 induces barrier dysfunction by downregulating keratinization and establishment of skin barriers. On the other hand, IL‐22 activates the innate immune response and increases antimicrobial peptides in AD lesional skin.

### 3.6 Differential Expression of Genes Involved in the Skin Barrier and Keratinization Induced by IL‐4 and IL‐13 Compared to IL‐22

**FIGURE 5:**
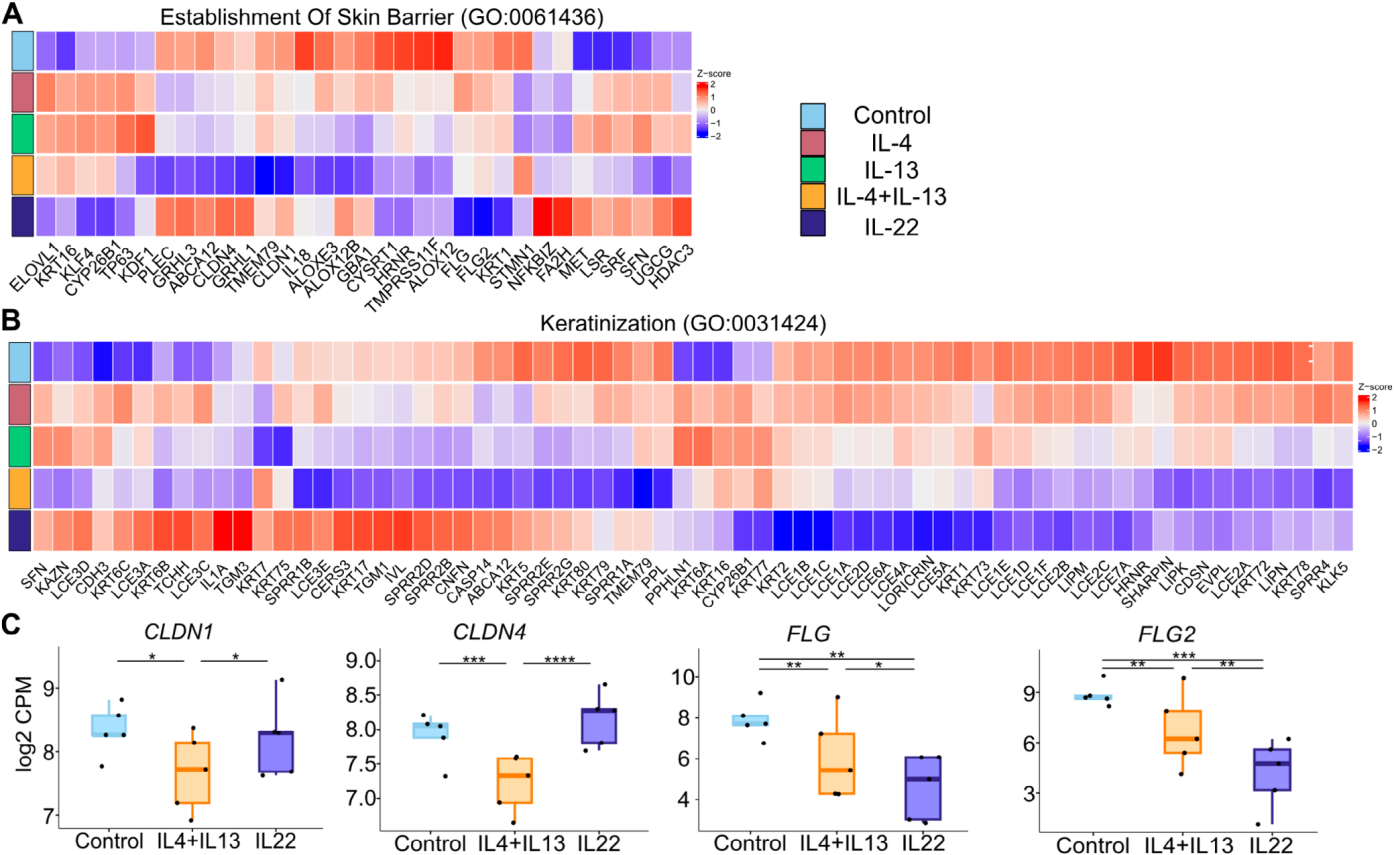
IL‐4, IL‐13, and IL‐22 modify the skin epithelial barrier and keratinization. (A, B) Heatmaps show the differentially expressed genes related to the skin barrier structure and function, and to keratinization. (C) Gene expression comparison of indicated epithelial barrier molecules in the control (CTRL), upon IL4 + IL13, and IL22 stimulation groups. *p < 0.05, **p < 0.01, ***p < 0.001, **** *p* < 0.0001.

RNA‐seq analysis was used to identify the differential gene expression profile in response to each cytokine (Figure 5). In addition to an inflammatory response, both type‐2 (IL‐4 and IL‐13) and IL‐22 stimulations impaired the epithelial barrier as indicated by the downregulation of genes including *CLDN1*, *FLG*, *FLG2*, *LORICRIN*, *LCEs*, and *KRT1* (Figure 5A,B), which play a role in the establishment of the skin barrier and keratinization pathways. Interestingly, we observed distinct differential expression of genes involved in the skin barrier function between the type‐2 cytokines (IL‐4 and IL‐13) and IL‐22. The combination of type‐2 cytokines strongly reduces the expression of *CLDN1* and *CLDN4*, although it is not affected by IL‐22 stimulation at 24 h. In contrast, the filaggrin genes *FLG* and *FLG2* are strongly downregulated by IL‐22 (Figure 5C). The type‐2 cytokines reduced the expression of certain genes, such as *KRT5*, *IVL*, and *CASP14*, while elevating the expression of *KRT16*, *KRT6A*, *KLF4*, and *ELOVL1*. Notably, IL‐22 did not alter the differential expression of these genes. Taken together, these findings demonstrate that the gene expression profile of genes involved in the skin barrier function and keratinization is distinct in samples stimulated by both IL‐4 and IL‐13 compared to IL‐22.

**FIGURE 6:**
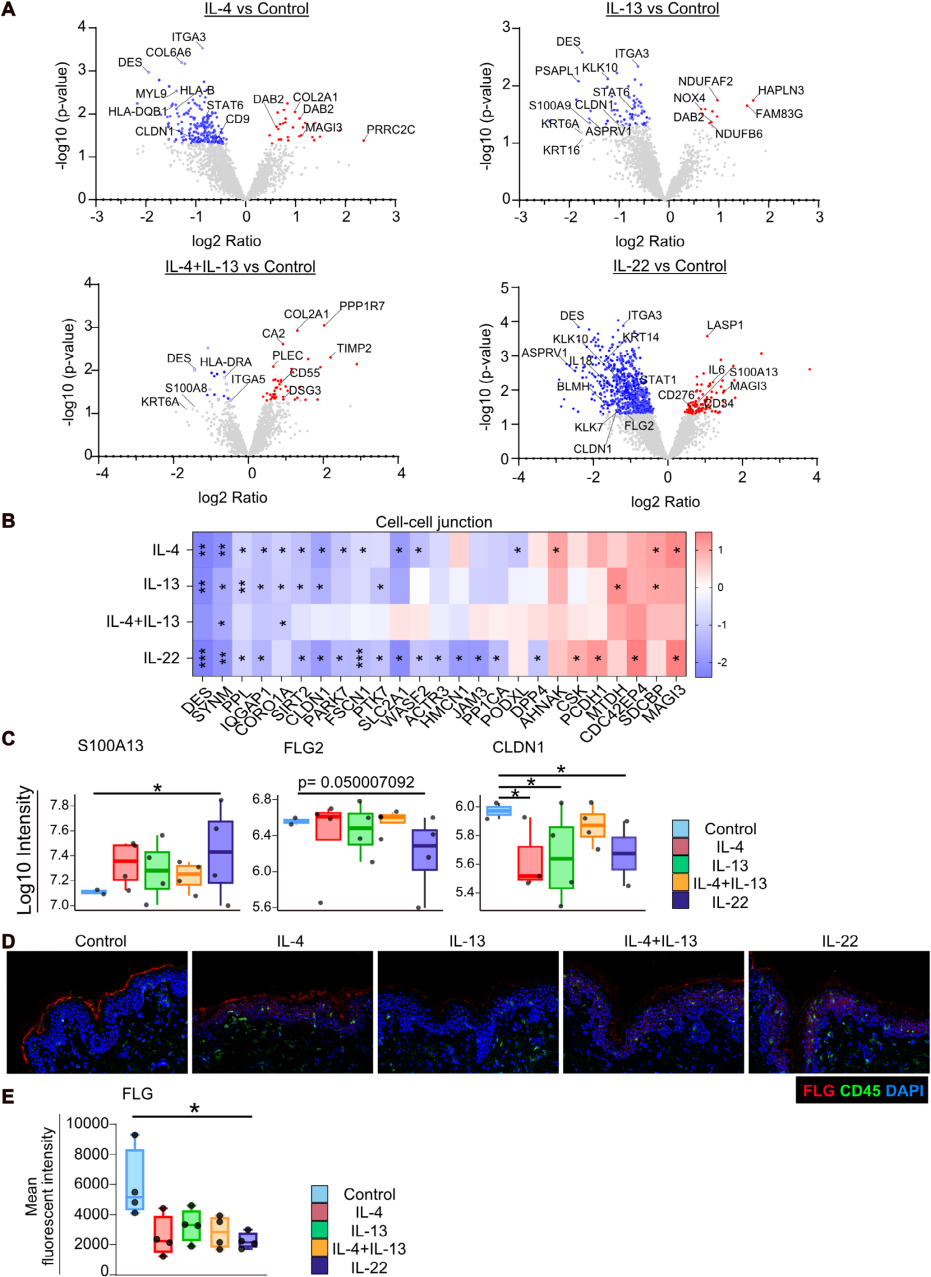
IL‐4, IL‐13, and IL‐22 decrease skin barrier‐related proteins. (A) Volcano plots show the differentially expressed proteins in comparison with the indicated groups compared to the control. Proteins with a *p* value of less than 0.05 are marked in red (Up) and blue (Down) (*n* = 3 biological donors). (B) Representative significant Gene Ontology terms of biological processes (GO BP) associated with the differentially expressed proteins. (C) Box plot of the intensities of the indicated proteins. Limma linear model, *p < 0.05. (D) Representative immunohistochemistry images of FLG (red), CD45 (green), and DAPI (blue). FLG expression was evaluated with CD45+ cells in the ex vivo skin upon the different stimulations (control, IL‐4, IL‐13, IL‐4 + IL‐13, and IL‐22; *n* = 4 biological donors). (E) Box plots comparing the mean fluorescent intensity of FLG expression. (*n* = 4), ONE way ANOVA, *p < 0.05.

To confirm the changes at the proteomic level, protein extract from skin samples taken 24 h after cytokine stimulation was analyzed using Orbitrap mass spectrometry. Proteins associated with cell–cell junctions were significantly suppressed by IL‐4, IL‐13, and IL‐22 (Figure 6A–C and Table S5), such as claudin‐1 (CLDN1) and desmin (DES). IL‐22 stimulation also impaired the expression of FLG2. DSG3 and membrane‐associated guanylate kinase, WW, and PDZ domain‐containing protein 3 (MAGI3) were upregulated by IL‐4, IL‐13, and IL‐22. In addition, IL‐13 and IL‐22 reduced aspartic peptidase retroviral‐like 1 (ASPRV1) expression, a protease involved in processing profilaggrin to filaggrin. In the context of filaggrin degradation by proteases, both kallikreins 7 and 10 (KLK7, KLK10) were downregulated by IL‐22, but only KLK10 by IL‐13 (Figure S4A). Interestingly, IL‐4 and IL‐13 decreased STAT6 expression, whereas IL‐22 suppressed STAT1 expression (Figure 6A), which might show negative feedback responses. The cytokine stimulations also altered the expression of S100A anti‐microbial proteins. S100A9 and S100A8 were downregulated by IL‐13 and by IL‐4 + IL‐13, respectively. In contrast, IL‐22 induced a significantly elevated expression of S100A13 (Figure 6C), in concordance with our spatial transcriptomics data.

Stimulation with IL‐4, IL‐13, IL‐4 + IL‐13, and IL‐22 reduced the expression of FLG in the skin (Figure 6D,E and Figure S5A). There are many CD45‐positive resident immune cells, and our findings in NativeSkin samples reflect the diverse and numerous immune responses occurring within the skin. Taken together, these results demonstrate the strong impact of IL‐4, IL‐13, and IL‐22 stimulation on CLDN1 and FLG expression after 24 h.

### 3.7 Dupilumab Restores IL4/IL13‐Signature Genes in Atopic Dermatitis Skin Lesions

**FIGURE 7:**
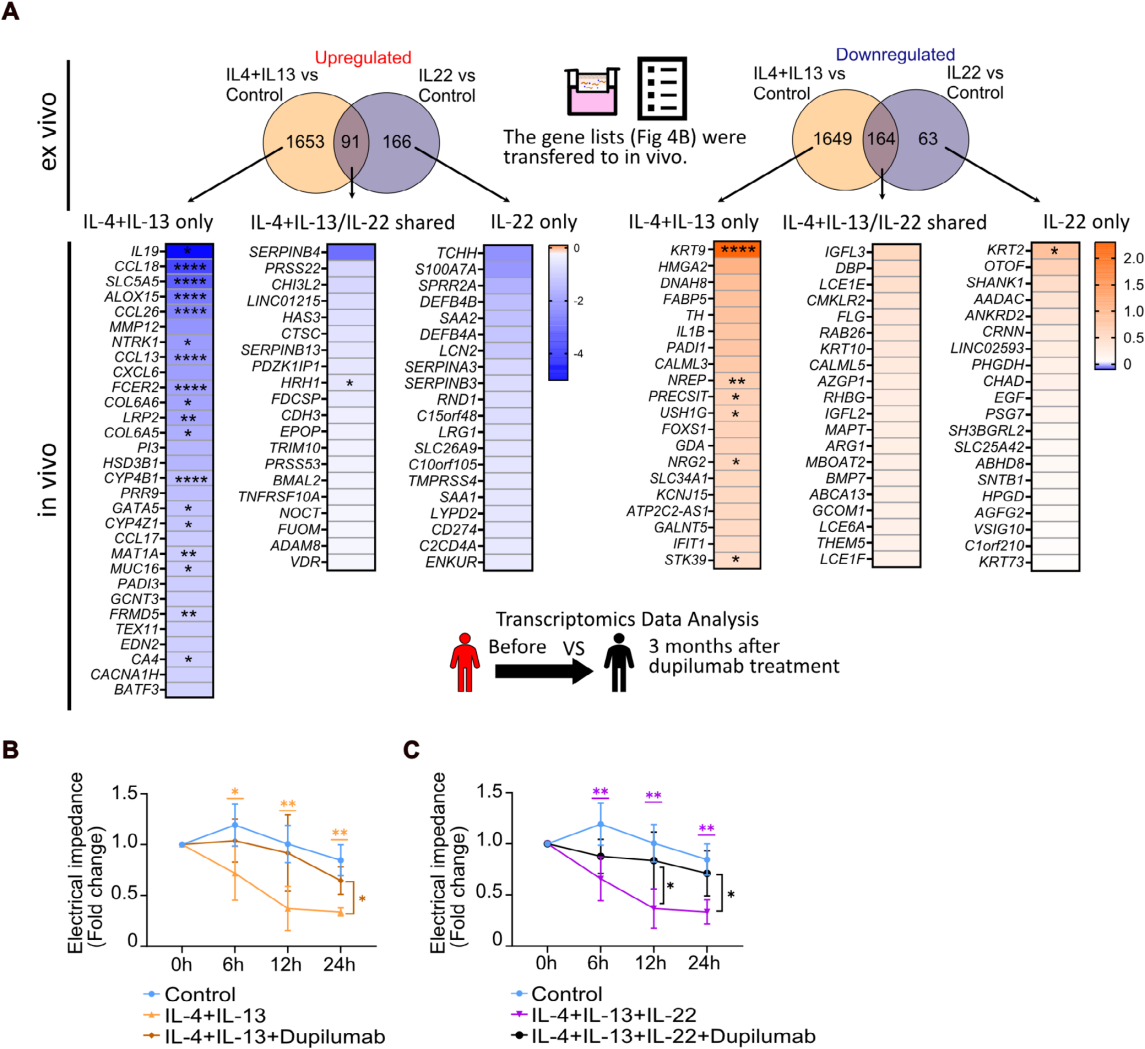
Blockade of IL4Rα restores gene expression induced by IL‐4 and IL‐13 but not by IL‐22. (A) Heatmaps showing the gene expression changes (log2 fold change) after 3 months of dupilumab treatment in lesional AD skin compared to before the treatment in the gene lists identified in Figure 4B. Highly regulated genes are shown. *p < 0.05, **p < 0.01, ***p < 0.001, ****p < 0.0001. FDR. (B, C) Ex vivo skins (*n* = 5 donors) were treated with IL‐4 + IL + 13 (100 ng/mL each) and with IL‐4 + IL‐13 + IL‐22 (100 ng/mL each) with or without dupilumab (100 μg/mL). Electrical impedance measurements were performed before and after treatment at indicated time points. The data were normalized with the impedance values at 0 h in each sample. A two‐way ANOVA test was used to compare differences between each stimulation and control at each time point. Data is shown as mean ± SEM. The colored stars indicate the statistical significance corresponding to the colors of the stimulations shown in the legend. *: *p* < 0.05, **: *p* < 0.01, **** *p* < 0.0001.

To analyze further, we investigated the effect of dupilumab on AD lesions with the data from GSE157194 [29] on the gene lists shown in Figure 4B and Table S4. In the former study, transcriptomic analyses of AD patient skin biopsies were performed after 3 months of dupilumab treatment. As shown in Figure 7A, many of the genes in the combined IL‐4 and IL‐13 stimulation (IL‐4 + IL‐13 only) were significantly suppressed in vivo by 3 months of dupilumab treatment. However, among the genes that were differentially expressed by stimulation with the combination of IL‐4 and IL‐13, and IL‐22 alone (IL‐4 + IL‐13/IL‐22 shared), only histamine receptor 1 (*HRH1*) was suppressed by dupilumab treatment. Moreover, dupilumab restored the expression of many genes suppressed by the type 2 cytokines (IL‐4 + IL‐13 only), including *KRT9*, *NREP*, *PRECSIT*, *USH1G*, *NRG2*, and *STK39*. However, only *KRT2* was upregulated by dupilumab treatment in IL‐22‐induced genes. A key skin barrier‐related gene, *FLG*, was not significantly restored after 3 months of dupilumab treatment. These results suggest distinct transcriptomic profiles in samples stimulated by IL‐4/IL‐13 only (type 2‐specific), IL‐4 + IL‐13/IL‐22 shared (namely, both IL‐4 and IL‐13, and IL‐22), and IL‐22 only (IL‐22‐specific). Type 2‐specific genes were rapidly restored after dupilumab treatment. In particular, gene expression of IL‐22 only and IL‐4 + IL‐13/IL‐22 shared genes persisted and were nonresponsive to therapy after 3 months.

### 3.8 Blockade of IL4Rα Prevented Epithelial Barrier Impairment Induced by IL‐4 and IL‐13

Based on the above results, we further investigated the effects of dupilumab in our ex vivo skin model. We stimulated ex vivo skins with the combination of the type‐2 cytokines (IL‐4 + IL‐13) and with IL‐4 + IL‐13 + IL‐22, treated with or without dupilumab (100 μg/mL). The concentration of dupilumab used was chosen based on previous studies [37, 38]. The ex vivo skins were incubated overnight with dupilumab upon arrival, followed by cytokine stimulation.

As seen in Figure 7B, IL‐4 + IL‐13 significantly lowered the electrical impedance at 6 h, 12 h, and 24 h. Pretreatment with dupilumab significantly reduced the decrease in the electrical impedance induced by the type‐2 cytokines after 24 h of stimulation (Figure 7B). To better understand AD pathogenesis, we further stimulated the ex vivo skins with type‐2 cytokines together with IL‐22. Treatment with dupilumab for 24 h restored impedance values even when the skin samples were exposed to the combination of IL‐4, IL‐13, and IL‐22 (Figure 7C). Taken together, these results demonstrate that dupilumab mitigates the cytokine‐induced inflammation and barrier impairment present in AD skin.

## 4 Discussion

The present study highlights the distinct roles of cytokines, IL‐4, IL‐13, and IL‐22 on the immune and inflammatory response of the skin and its barrier integrity using ex vivo human skin. We identified key genes and enriched pathways involved in these events, including skin barrier establishment, epidermal differentiation, antimicrobial peptides, angiogenesis, and neurogenesis. These findings provide deeper insights into the molecular mechanisms underlying AD, paving the way for novel treatment strategies with new potential therapeutic targets and biomarkers.

Ex vivo skin models provide a more comprehensive overview of the effect of AD‐related cytokines on the complex immune responses in the human skin than reconstructed human skin. The role of IL‐22 and the type‐2 inflammatory cytokines IL‐4 and IL‐13 in AD pathogenesis is well‐known [12, 14, 15]. However, the interplay between these cytokines, epidermis, dermis, appendages, and resident cells within the human skin is not fully understood. In this study, we stimulated our model with equal concentrations of IL‐4, IL‐13, and IL‐22 to ensure balanced and consistent pathway activation, allowing for direct comparison of their effects. However, it is important to note that the actual levels of these cytokines in the skin of patients with AD are not equal—IL‐13 is typically present at higher levels than IL‐4 in lesional skin. Despite its lower abundance, IL‐4 plays a crucial role in AD pathogenesis by initiating Th2‐driven inflammation and skin barrier disruption [39, 40]. Notably, mechanical scratching alone can induce IL‐4 expression [41], and IL‐4 levels are elevated in lesional AD skin compared to non‐lesional AD skin and psoriasis skin [42]. EIS has been recently described as a useful tool for quantifying epidermal barrier integrity rapidly and unobtrusively in children, adults, and experimental mouse models [21, 22, 23, 43]. Using a multi‐omics approach, we address the role of these cytokines in disrupting the epithelial barrier function in recently developed ex vivo skin models [23, 36].

Transcriptomic analysis showed the precise spatial expression of essential skin barrier molecules. AD lesions had a reduced expression of *CLDN1*, *CLDN4*, *FLG*, and *FLG2* in the epidermis. On the other hand, the expressions of desmosomes, such as *DSG1* and *DSG3*, and antimicrobial peptides, such as *S100A7*, *S100A8*, and *S100A9*, were significantly elevated in the lesional skin of AD patients. In contrast, we demonstrate that *CLDN5*, *CLDN7*, *CLDN8*, *OCLN*, *TJP1*, *TJP2*, and *S100A13* were not downregulated in the epidermis and were expressed more in the dermis. It is important to emphasize that our ex vivo skin inflammation model shown here is representative of the in vivo situation of the inflammatory and skin barrier‐damaging pathogenetic events in AD. We demonstrated that all three stimulations reduced the impedance values, as observed in lesioned human AD skin.^22^ The stimulation with type 2 cytokines and IL‐22 significantly suppressed several key epithelial barrier genes including keratin genes, such as *KRT1*, *KRT5*, and *KRT17*, as well as FLG and *FLG2*.

The defective epithelial barrier in AD skin facilitates the invasion by opportunistic pathogen bacteria. Antimicrobial peptides play a protective role by directly killing the microorganisms on the epithelial surface. As expected, genes related to antimicrobial peptides were upregulated in AD lesional skin. However, type‐2 cytokines and IL‐22 showed distinct effects on the antimicrobial response in NativeSkin. IL‐13 downregulates *S100A7*, *S100A8*, *S100A9*, *TLR2*, *AGER*, and *HMGB1*. TLR2 and AGER (known as RAGE) are directly activated by S100 proteins, aggravating inflammatory responses. In addition, HMGB1 can form complexes with S100 proteins and augment their inflammatory potential. Although type‐2 cytokines downregulated antimicrobial peptides, IL‐22 was a key cytokine in increasing their mRNA and protein expression. These findings indicate an important role for IL‐22 in preventing bacterial colonization in the skin [44].

Stimulations with IL‐4 and IL‐13 alone, and the combined cytokines in the ex vivo human skin model significantly upregulated genes well known to be related to type‐2 inflammation, such as *CCL13*, *CCL17*, *CCL26*, *SOCS1*, *POSTN*, *IL13RA2*, and *GATA3*. In addition, we identified *CAPN14*, *LURAP1L*, *DUOX1*, *DUOX2*, *DUOXA1*, *DUOXA2*, and *NTRK1* as highly type‐2‐related genes. These findings provide insights into the genes that may be involved in the pathogenesis of AD. Calpain‐14 (CAPN14), a calcium‐dependent cysteine protease, is overexpressed in the epithelium of patients with eosinophilic esophagitis (EoE), suppressing DSG1 expression and consequently impairing the epithelial barrier function [45, 46]. Leucine‐rich adaptor protein 1 like (LURAP1L) is an activator of the NF‐kB pathway that drives the production of pro‐inflammatory cytokines [47] and was also found to be elevated in samples stimulated by type‐2 cytokines. Dual oxidases (DUOX) 1 and 2 are NADPH oxidases that generate H_2_O_2_. We have also observed the upregulation of neurotrophic receptor tyrosine kinase 1 (NTRK1), which is a high‐affinity receptor for nerve growth factor (NGF) and critical for the growth of sensory neurons. In a study of eosinophilic esophagitis, IL‐13 induced NTRK1 expression and their levels were correlated with disease activity [48]. Our findings suggest that IL‐4 and IL‐13 enhance epithelial cell responsiveness to NGF by regulating NTRK1 levels, which likely contributes to itchiness in AD.

Furthermore, we identified significant differences in the regulation of angiogenesis, namely IL‐13 downregulated angiogenesis and vascular development pathways; however, additional IL‐4 stimulation significantly upregulated these genes, such as *FLT1*, *PDGFRA*, *PDGFRB*, *PECAM1*, *TIE1*, and *TEK*. This suggests that IL‐4 plays a more prominent role in promoting vascular remodeling in the inflammatory skin microenvironment.

IL‐22, a cytokine belonging to the IL‐20 subfamily, is reported to be upregulated in patients with AD. By comparing IL‐4, we demonstrated that IL‐13 significantly downregulated IL22RA1 expression, potentially suppressing IL‐22 signaling. Conversely, IL‐22 upregulated IL4R expression. The expression of IL10RB, which composes the heterodimer of the IL‐22 receptor, was not regulated by each cytokine stimulation. Interestingly, the IL4Rα inhibitor, dupilumab, activates IL‐22 inflammation in T cell oligoclones, which is characterized by head and neck dermatitis [49]. These results suggest that type‐2 stimulation, especially IL‐13, tries to suppress IL22 signaling and the innate immune defense. As shown in Figure 4, IL‐22 also significantly impairs epithelial barrier integrity in human skin. The enriched pathways in the downregulated genes by IL‐22 are related to keratinization, establishment of the skin barrier, epidermal cell differentiation, and cell–cell adhesion including *FLG*, *FLG2*, *LORICRIN*, *DSG1*, *KRT1*, and *CLDN8*, similarly to the stimulation combining IL‐4 and IL‐13. The upregulated genes induced by IL‐22 are different from those from type‐2 stimulation. These include genes related to an innate immune response, NF‐kappaB signaling, T‐helper cell differentiation, epidermal growth factor receptor signaling, and positive regulation of epithelial cell proliferation pathways. In this study, we identified an IL‐22‐specific cluster including upregulating genes, such as *IL4R*, *STAT3*, *CCL4*, *CXCL16*, *S100A7A*, *DEFB4A*, *SERPINA1*, *SERPINB1*, and downregulated genes, such as *KRT2*, *KLF4*, *KRT73*, *IL36B*, and *SPINK5*. Type‐2 cytokines and IL‐22 induce different types of inflammation and expression of antimicrobial peptides. Previous studies have demonstrated IL‐22 as a main driver of epidermal hyperplasia, antimicrobial peptide production, and barrier defects by upregulating keratinocyte differentiation and inhibiting terminal differentiation [50, 51]. The efficacy and safety of the IL‐22 monoclonal antibody fezakinumab have been evaluated in adults with moderate‐to‐severe AD, uncontrolled by conventional treatments [12]. The unique biomarkers corresponding to IL‐22 inflammation are of clinical value. We identified the differentially expressed genes in IL‐22 stimulated samples and found that these were not significantly altered after 3 months of dupilumab treatment. Importantly, *FLG* is downregulated by both type‐2 cytokines and IL‐22 indicating that *FLG* was not significantly restored by only blocking the type‐2 pathway. In addition, *SERPINB4*, known as *SCCA2*, was identified as a common gene in the skins stimulated with IL‐4, IL‐13, and with IL‐22. SCCA2 has been evaluated as a biomarker for the clinical severity of AD according to EASI [52, 53]. The requirement for dual blocking of IL‐4 and IL‐13 to suppress type 2 inflammation has been previously reported in primary cell assays and house‐dust mite‐induced asthma mouse model [38]. We identified genes regulated by only the type‐2 pathway with a good response to dupilumab after 3 months of treatment. Genes regulated by both type‐2 cytokines and IL‐22, and unique genes only induced by IL‐22 were not responsive to anti‐IL4Rα treatment. This data offers a stratification of the unique signatures of type‐2 signaling and IL‐22, providing insights into their distinct roles. On the other hand, it is reported that 16 weeks of dupilumab treatment reduced serum IL‐22 levels and keratosis [54]. Dupilumab may effectively reduce systemic IL‐22 levels and improve clinical symptoms, but IL‐22‐related gene expression in skin tissue may persist due to local cytokine signaling and tissue‐specific responses that are not fully suppressed by IL‐4Rα blockade. These findings have important clinical implications for personalized treatment strategies in AD. Identifying biomarkers that distinguish IL‐22‐predominant inflammation, such as S100As and DEFB4A, could aid in stratifying patients. Future clinical studies incorporating IL‐22‐related biomarkers could enable more precise, mechanism‐based interventions for patients with varying inflammatory signatures. In this study, dupilumab restores electrical impedance in an ex vivo skin model exposed to IL‐4, IL‐13, and IL‐22, demonstrating its ability to preserve skin barrier function in AD. While the mechanism by which dupilumab reverses the reduction in electrical impedance remains unclear, our data demonstrate that IL‐22 upregulates IL‐4Rα expression. We also observed the upregulation of pathways related to the innate immune response, T‐helper cell differentiation, and T‐cell activation, as well as the downregulation of pathways involved in keratinization and skin barrier formation—pathways that are also affected by the combination of IL‐4 and IL‐13 (Figure 4C). These findings suggest a potential cross‐talk between type 2 cytokines and IL‐22 signaling pathway.

In the present study, we observed that both the type‐2 and IL‐22 cytokines generally suppressed the protein expression of cell differentiation and barrier‐related molecules. The expression of CLDN1 and DES was downregulated by type‐2 cytokines and IL‐22. In addition, FLG2 was also downregulated by IL‐22 stimulation. Interestingly, IL‐13 and IL‐22 also suppressed the expression of ASPRV1, a key protease for the processing of pro‐filaggrin to filaggrin, highlighting the important role of these cytokines on healthy skin barrier homeostasis and the keratinocyte differentiation processes. Kallikrein‐related peptidases (KLKs) are epidermal proteins involved in skin desquamation and inflammation. The downregulation of KLK7 and KLK10 might represent a counter‐reaction to the reduced expression of FLG. Therefore, these findings suggest that IL‐4, IL‐13, and IL‐22 induce skin barrier dysfunction in AD by suppressing FLG expression. Interestingly, type 2 cytokine stimulation significantly downregulated STAT6 expression, while IL‐22 significantly downregulated STAT1 expression. These findings highlight the distinct negative feedback control under type‐2 and IL‐22 inflammation in the skin.

Although our ex vivo skin models treated with type‐2 cytokines and IL‐22 provide new insights into the skin responses to AD‐related cytokines, they do have some limitations. First, the concentrations of cytokines used in our experiments do not fully reflect the heterogeneous and complex cytokine environment present in AD lesional skin. Differences in cytokine abundance may influence the biological responses observed in vivo. Second, our ex vivo skin samples were primarily obtained from female donors undergoing plastic surgery, introducing a potential sex bias and limiting the generalizability. Third, we performed bulk RNA‐seq in our ex vivo skin samples to capture global gene expression changes, which do not allow spatial gene expression patterns. Additionally, our study focused on acute cytokine stimulation and did not assess the long‐term or dose‐dependent effects of each cytokine. Future research incorporating spatial transcriptomics, with a more diverse donor pool, and extended experimental timelines will be essential to address these limitations.

In conclusion, the present study demonstrates distinct differences in response to IL‐4, IL‐13, and IL‐22 signaling in human skin at the transcriptome and proteome levels. Overall, our data indicate that IL‐4, IL‐13, and IL‐22 cytokines impair skin barrier integrity, especially by downregulating CLDN1 and FLG expression. In addition, IL‐4 induces cell adhesion and angiogenesis, IL‐13 drives the downregulation of IL‐22 signaling, and IL‐22 promotes an innate immune response including antimicrobial peptide production, as well as the upregulation of IL4R, creating a feedback loop that contributes to the type‐2 signaling. Clarifying the inflammatory mechanisms triggered by AD‐related cytokines may be important for developing new treatments for allergic diseases, as barrier dysfunction plays a critical role in their pathogenesis. Personalized medicine requires the identification of biomarkers for targeted treatment, and through this study, we provide a better understanding of the role of IL‐4, IL‐13, and IL‐22 in the human skin.

## Author Contributions

Conceptualization: Cezmi A. Akdis and Yasutaka Mitamura. Methodology: Paolo D'Avino, Manru Li, Philipp Gessner, Patrick Westermann, Carina Beha, Yagız Pat, and Yasutaka Mitamura. Investigation: Paolo D'Avino, Christoph Messner, and Yasutaka Mitamura. Visualization: Paolo D'Avino, Juno Kim, Christoph Messner, and Yasutaka Mitamura. Funding acquisition: Cezmi A. Akdis. Project administration: Paolo D'Avino and Yasutaka Mitamura. Supervision: Yagiz Pat, Claudia Traidl‐Hoffmann, Nicolas Gaudenzio, Jeremy Bost, Christoph Messner, Cezmi A. Akdis, and Yasutaka Mitamura. Writing – original draft: Paolo D'Avino and Yasutaka Mitamura. Writing – review and editing: Cezmi A. Akdis and Yasutaka Mitamura.

## Conflicts of Interest

C.A.A. reports a patent application on “Methods and medical devices for analyzing epithelial barrier function.” J.B., C.A.A., and Y.M. report a patent for the technology titled “Methods and apparatus for measuring electrical impedance and assessing biological conditions of tissue samples.” J.B. declares receiving personal fees from SciBase AB. N.G. declares receiving personal fees from Genoskin SAS. C.A.A. has received research grants from the Swiss National Science Foundation, European Union (EU CURE, EU SynAir‐G), Novartis Research Institutes (Basel, Switzerland), Stanford University (Redwood City, Calif), and SciBase (Stockholm, Sweden); is the Co‐Chair for EAACI Guidelines on Environmental Science in Allergic diseases and Asthma; is on the Advisory Boards of Sanofi/Regeneron (Bern, Switzerland, New York, USA), Stanford University Sean Parker Asthma Allergy Center (CA, USA), Novartis (Basel, Switzerland), Glaxo Smith Kline (Zurich, Switzerland), Bristol‐Myers Squibb (New York, USA), Seed Health (Boston, USA), and SciBase (Stockholm, Sweden); and is the Editor‐in‐Chief of Allergy. P.D., J.K., M.L., P.G., P.W., C.B., Y.P., C.T.‐H., and C.B.M. have nothing to declare within the scope of this work.

## Supporting information


**Data S1:** all70060‐sup‐0001‐DataS1.zip.

## Data Availability

The data that support the findings of this study are available from Gene Expression Omnibus (GEO): GSE292848. Restrictions apply to the availability of these data, which were used under license for this study. Data are available from the author(s) with the permission of Gene Expression Omnibus (GEO): GSE292848. The accession number for the raw and processed sequencing data reported in this paper is Gene Expression Omnibus (GEO): GSE292848 (RNA sequencing in ex vivo human skin), GSE157194 (Möbus et al. 2021), and GSE197023 (Mitamura et al. 2023). Analysis scripts for RNA‐seq analyses are available at https://gitlab.com/juno‐kim/nativeskin‐cytokine.
