# Supplementary material for: Distinct Roles of IL‐4, IL‐13, and IL‐22 in Human Skin Barrier Dysfunction and Atopic Dermatitis
Source: Allergy. 2025 Sep 23;81(2):480–97. doi: 10.1111/all.70060 (PMC12862561; doi:10.1111/all.70060)
Supplement: Supplementary file 1 — Data S1: all70060‐sup‐0001‐DataS1.zip. [file ALL-81-480-s001.zip › all70060-sup-0001-AppendixS1.docx]

**Supplementary Methods**

Title: Distinct roles of IL-4, IL-13, and IL-22 in human skin barrier dysfunction and atopic dermatitis

## Cytokine treatment on ex-vivo human skin: source, conditions, and sampling methods

Ex-vivo human skin samples (NativeSkin®) were purchased from Genoskin SAS (www.genoskin.com). Anonymized human skin samples were obtained from donors who underwent abdominoplasty procedures and had given their written informed consent. Donors

had no record of allergies or dermatological disorders and did not use corticosteroids. Full ethical approval for the study protocol was obtained from the French ethical research committee (Comité de Protection des Personnes), and authorization was granted by the French Ministry of Research. Adult human skin explants were obtained from ten healthy female donors (table 1) and bio-stabilized. All samples were provided as 15 mm diameter round natural human skin biopsies in proprietary custom-built plastic inserts. These natural human skin samples were cultured in 2-2.5 mL of chemically defined xeno-free Genoskin medium at 37°C in a standard incubator (5% CO_2_).

Ex-vivo human skin samples were treated with IL-4 (100 ng/mL), IL-13 (100 ng/mL), IL-4+IL-13 (100 ng/mL each), IL-22 (100 ng/mL), dupilumab (100 µg/mL), or PBS with 0.1% BSA. The media was exchanged after 12 h of stimulation. The skin samples were collected after 24 hours of stimulation and split into pieces. Each piece was stored in RNAlater (Agilent, USA) for RNA-seq, snap frozen for proteomics analyses, and embedded in Tissue-Tek® O.C.T. Compound (Sakura Finetek, USA) for immunohistochemistry (IHC) experiment respectively.

## EIS measurements

EIS measurements were performed using Nevisense® (SciBase, Sweden) according to a previous study ^22^. EIS measures a material's opposition to the flow of alternating currents at various frequencies. Specifically, tissue EIS values can reflect the pathophysiological status of the tissue. The electrical impedance was measured at 35 different frequencies distributed between 1 kHz and 2.5 MHz at two depths in three permutations. EIS measurements were performed at 0, 6, 12, and 24 h after cytokine treatment. Measurements were made in triplicates at each time point.

## Spatial RNA sequencing

The spatial RNA-seq data used in this study was derived from our recent publication^1^. Skin tissues were obtained from 6 healthy control donors (aged 24-63 years, 2 males and 4 females) and 7 AD patients (aged 24-63 years, 4 males and 3 females, mean SCORAD: 44.49 ± 13.73). For further details, see the previous manuscript.

The previously published spatially resolved transcriptomics (SRT) data of human skin biopsy samples was obtained from GEO under the accession number GSE197023. The analysis was conducted again in R with Seurat (version 4.4.0)^2^. Spots were filtered according to the following criteria: (1) unique molecular identifiers ≤ 200, (2) unique genes ≤ 100, (3) ≥ 20% mitochondrial gene content (4) ≥ 10% genes coding for hemoglobin proteins. Samples resulting in a total spot count of < 100 were entirely removed, in order to improve sample integration. Furthermore, genes coding for mitochondrial, ribosomal, and hemoglobin proteins were removed. The data was log-normalized and integrated by identifying 5,000 integration anchors using reciprocal principal component analysis (RPCA) with a k.anchor of 10 and including all common genes across all samples. The integrated data underwent another PCA and the first 13 principal components were considered for identifying the k-nearest neighbors. Clustering was performed using the Louvain algorithm with a resolution parameter of 0.1. The clusters were systematically annotated by checking the top 10 markers (differentially expressed genes) identified in each cluster by FindAllMarkers against the Human Protein Atlas tissue cell type database^3^. Differential expression analysis between tissue conditions in selected anatomical regions, i.e. upper and lower epidermis, which were identified by unsupervised clustering, was conducted using Seurat’s built-in function FindMarkers. Each spot of the SRT data was assumed to consist of multiple different cell types and were therefore regarded as miniature bulk RNA-seq samples. The Wilcoxon rank-sum test was used to control the type I error rates in very large-sample-size RNA-seq experiments. The p-values were adjusted by Bonferroni correction.

## Bulk RNA-seq in ex-vivo human skin

One-eighth of 15 mm diameter ex-vivo human skin was minced and digested in Trizol and processed with Precellys devices (Precellys 24 homogenizer) at 4°C to extract RNA from the tissue. The RNA was then purified using an RNAeasy Plus Micro Kit (Qiagen) following the manufacturer’s guidelines. The concentration and quality of RNA were determined in each sample using a NanoDrop (Thermo Fisher Scientific) and 2200 TapeStation Automated Electrophoresis System (Agilent Technologies), and samples with RNA integrity numbers greater than 6.0 were chosen for sequencing. RNA library preparation and sequencing of samples were performed in two batches. Following the first round of sequencing with n=3, we performed a second round to increase the sample size. The libraries of the first batch were prepared using the Illumina Stranded mRNA Prep protocol and sequenced on the NovaSeq 6000 (Illumina, San Diego, CA) in a single read 100 bp configuration. Libraries of the second batch were prepared using the same protocol but were sequenced on a NovaSeq X Plus in a paired-end 150 bp configuration. In downstream processing, only the R1 files of the samples sequenced in the paired-end configuration were combined with the first single-end batch. The raw reads were processed using Cutadapt (version 2.5)^4^. Processed reads were aligned to the Ensembl GRCh38 human genome assembly and annotated with the Ensembl GRCh38.109 gtf file using STAR (version 2.7.9a)^5^. Exons were counted using the featureCounts read summarization function from the Subread package (version 2.0.1)^6^.

All analyses, including those performed on already published data sets, were performed in the R programming language (version 4.4.1)^7^. The dupilumab-treated human skin RNA-Seq data was obtained from Gene Expression Omnibus (GEO) under the accession number GSE157194^8^. Differential gene expression analysis was performed by fitting negative binomial generalized linear models followed by quasi-likelihood F-tests using edgeR (version 4.2.1)^9^. In the native skin samples, the linear model accounts for the biological variation between different donors. In the dupilumab-treated human skin data, a paired design was used in order to account for the differences in the effect on each individual patient. Enrichment analysis for gene ontology was performed in topGO (version 2.56.0)^10^, using the Elim algorithm combined with Fisher’s exact test. All heatmaps were generated using ComplexHeatmap (version 2.20.0) ^11^.

## Sample Processing for LC-MS

Ex-vivo skins were disrupted in RIPA Lysis buffer (1:10, EMD Millipore Sigma-Aldrich, MA, USA) with protease inhibitor cocktail tablets (cOmplete Tablets, Mini EDTA-free, EASYpack, Roche, Switzerland). Afterward, ex-vivo skins were homogenized using CK-Mix beads (Bertin, France) and the Precellys 24 homogenizer. Skin sections were then processed with 2 cycles of 6500 rpm for 20 seconds. The protein concentrations of tissues were measured by Pierce™ BCA Protein Assay Kits (Thermo Fisher Scientific, MA, USA). Samples were subsequently normalized to a concentration of 0.5 mg/mL with the 1X RIPA lysis buffer.
Samples were prepared according to the SP4 protocols^45^.

50 µL protein lysates with 0.5 µg/µL were thawed at RT. The protein lysates were sonicated for 5 min in an Elmasonic P 60 H bath sonicator (Elma Schmidbauer GmbH). 12.5 µL of 100 mM tris(2-carboxyethyl)phosphine (TCEP) / 400 mM chloroacetamide (CAA) mixture was pipetted to the lysates and the samples incubated for 5 min at 95 °C and 500 rpm (Eppendorf Thermomixer C). The samples were cooled down for 10 min at RT. The proteins were precipitated by adding 250 µL of acetonitrile. The plate was mixed for 5 s at 400 rpm. The plate was centrifuged for 10 min at 4000 rpm (Thermo Scientific Multifuge X3R). 280 µL of the supernatant was removed and the precipitate was washed three times with 200 µL of 80% ethanol (centrifugation for 5 min at 4000 rpm). Finally, 220 µL of the supernatant was removed and 140 µL 100 mM ABC (pH 8.0) was added to the wells; the plate was sonicated for 5 min in an Elmasonic P 60 H bath sonicator. Then, 10 µL of 0.05 µg/µL Trypsin/Lys-C was added to the wells and the plate was incubated for 18 h at 37 °C and 500 rpm. The digestion was stopped by the addition of 16.7 µL 10% formic acid. The peptides were desalted using an Oasis PRiME HLB 96-well μElution Plate. Samples were loaded onto the plate, the wells were washed twice with 400 μl 0.1% formic acid and once with 200 μl ddH_2_O, and eluted with 50 μl 70% acetonitrile after a 1-min incubation at room temperature. The liquid was evaporated with a SPD120 SpeedVac (Thermo Fisher Scientific), and samples were resuspended in 30 μl 3% acetonitrile and 0.1% formic acid by shaking for 10 min at 1000 rpm, sonicated for 5 min in an Elmasonic P 60 H bath sonicator.

## LC-MS measurements

Samples were acquired on an EASY-nLC1200 coupled to a Thermo Orbitrap Eclipse Tribrid MS in data-independent acquisition (DIA) mode. Buffer A consisted of 0.1% formic acid in water and buffer B 0.1% formic acid in 80% acetonitrile. A nanoLC column with an integrated emitter from CoAnn Technologies was used with the following dimensions: 75 μm ID x 25 cm L x 365 μm OD, ReproSil-Pur120 C18 particles (1.9 μm). An Acclaim PepMap 100 C18 pre-column was used (0.1 mm ID x 150 mm L with 5 μm particle size). Flow rate was set to 400 nL/min, and the following gradient was applied: 30 s from 0% B to 3% B, 30 s from 3% B to 6% B, 27.5 min from 6% B to 21% B, 10.5 min from 21% B to 31% B, 6 min from 31% B to 44% B, 3 min from 44% B to 100% B and a final wash of 7 min at 100% B. For the MS1 scan, orbitrap resolution was set to 120,000, with quadrupole isolation turned on. A scan range of 380 to 980 m/z was applied, the RF lens was set to 30%, and standard automatic gain control (AGC) target with a custom maximum injection time of 50 ms was applied. For the MS2 scan, orbitrap resolution was set to 15,000, with 50 fixed windows (12 m/z isolation window and 0.5 m/z overlap). Higher-energy collisional dissociation collision energy was fixed at 30%. MS/MS scan range was defined as 145 to 1450 m/z, and RF Lens was set to 30%. AGC Target was set to 1000% with a custom maximum injection time of 22 ms.

## Proteomic data analysis

Raw data was processed using DIA-NN software with default settings ^46^. The search was performed library-free with in-silico digestion and deep learning–based spectra and retention time prediction. The human fasta file was downloaded from UniProt (3AUP000005640, downloaded on 19 January 2023). Downstream analysis was performed with R. For protein analysis, PG. Normalised values were used. Precursors were filtered for proteotypicity, precursor q-values were filtered with a threshold of 0.01, and protein group q-values were filtered with a threshold of 0.05. Differential expression analysis was performed using the limma R package, incorporating the donors into the models^47^ . The linear models were fitted protein-wise using the lmFit function within the limma package. The t-statistics were computed using the eBayes function, allowing for an intensity trend in the prior variance.

## Immunohistochemistry staining

Frozen embedded ex-vivo skin in Tissue-Tek® O.C.T. Compound (4 donors, 5 different conditions; control, IL-4, IL-13, IL-4+IL-13, IL-22 treated ex-vivo skin) were cut into 7 µm sections. The sections were fixed with 4% paraformaldehyde (Fluka, St Louis, Mo). Permeabilization and blocking were accomplished by incubating a mixture of 10% goat serum (DakoCytomation, Glostrup, Denmark), PBS containing 0.2% Triton X (Acros Organics, Geel, Belgium), and 1% BSA (Sigma-Aldrich) in PBS. Specimens were then stained with an anti-FLG antibody (Abcam, ab218395, mouse IgG1, 1:200), and an anti-CD45 antibody (Invitrogen, MA5-17687, rat IgG2A, 1:250). Slides were mounted with Fluoromount Aqueous Mounting Medium (Sigma-Aldrich) and DAPI (Sigma-Aldrich). Specimens were examined under a Zeiss LSM 780 (Carl Zeiss Microscopy GmbH, Oberkochen, Germany) in the tail mode scan, acquiring 6 pictures (3 vertical and 2 horizontal ones) at the same time. 10 squares with the same sizes were applied to each picture per all the donors for each condition. Statistical analysis was performed with the mean intensity calculated by FIJI (ImageJ). For a total of 40 squares of the same size for control, IL-4, IL-13, IL-4+IL-13, IL-22 were compared. For each picture, the background was calculated as well with the same size of square and then subtracted to the mean intensity value. One-way ANOVA was applied with adjusted p-values.

1. Mitamura, Y. *et al.* Spatial transcriptomics combined with single-cell RNA-sequencing unravels the complex inflammatory cell network in atopic dermatitis. *Allergy* **Online ahead print** (2023).

2. Hao, Y. *et al.* Integrated analysis of multimodal single-cell data. *Cell* **184**, 3573-3587.e3529 (2021).

3. Dusart, P. A tissue centric atlas of cell type transcriptome

enrichment signatures. In: Öling, S., Struck, E., Norreen-Thorsen, M.,, Zwahlen, M., von Feilitzen, K., Oksvold, P., Bosic, M., Iglesias, M.J., Renne, & T., O., J., Pontén, F., Lindskog, C., Uhlén, M., Butler, L.M., editors.: bioRxiv; 2023.

4. Martin, M. Cutadapt removes adapter sequences from high-throughput

sequencing reads.: EMBnet.journal; 2011.

5. Dobin, A. *et al.* STAR: ultrafast universal RNA-seq aligner. *Bioinformatics* **29**, 15-21 (2013).

6. Liao, Y., Smyth, G.K. & Shi, W. featureCounts: an efficient general purpose program for assigning sequence reads to genomic features. *Bioinformatics* **30**, 923-930 (2014).

7. Team, R.C. R: A Language and Environment for Statistical

Computing. R Foundation for Statistical Computing. 2024.

8. Möbus, L. *et al.* Atopic dermatitis displays stable and dynamic skin transcriptome signatures. *J Allergy Clin Immunol* **147**, 213-223 (2021).

9. Chen, Y. edgeR 4.0: powerful differential analysis of

sequencing data with expanded functionality and improved support for small

counts and larger datasets. In: Chen, L., Lun, A.T.L., Baldoni, P.L., Smyth, G.K., editor.: bioRxiv; 2024.

10. Alexa, A., Rahnenführer, J. & Lengauer, T. Improved scoring of functional groups from gene expression data by decorrelating GO graph structure. *Bioinformatics* **22**, 1600-1607 (2006).

11. Gu, Z., Eils, R. & Schlesner, M. Complex heatmaps reveal patterns and correlations in multidimensional genomic data. *Bioinformatics* **32**, 2847-2849 (2016).
