# Supplementary material for: Distinct Roles of IL‐4, IL‐13, and IL‐22 in Human Skin Barrier Dysfunction and Atopic Dermatitis
Source: Allergy. 2025 Sep 23;81(2):480–97. doi: 10.1111/all.70060 (PMC12862561; doi:10.1111/all.70060)
Supplement: Supplementary file 1 — Data S1: all70060‐sup‐0001‐DataS1.zip. [file ALL-81-480-s001.zip › all70060-sup-0002-AppendixS2.docx]

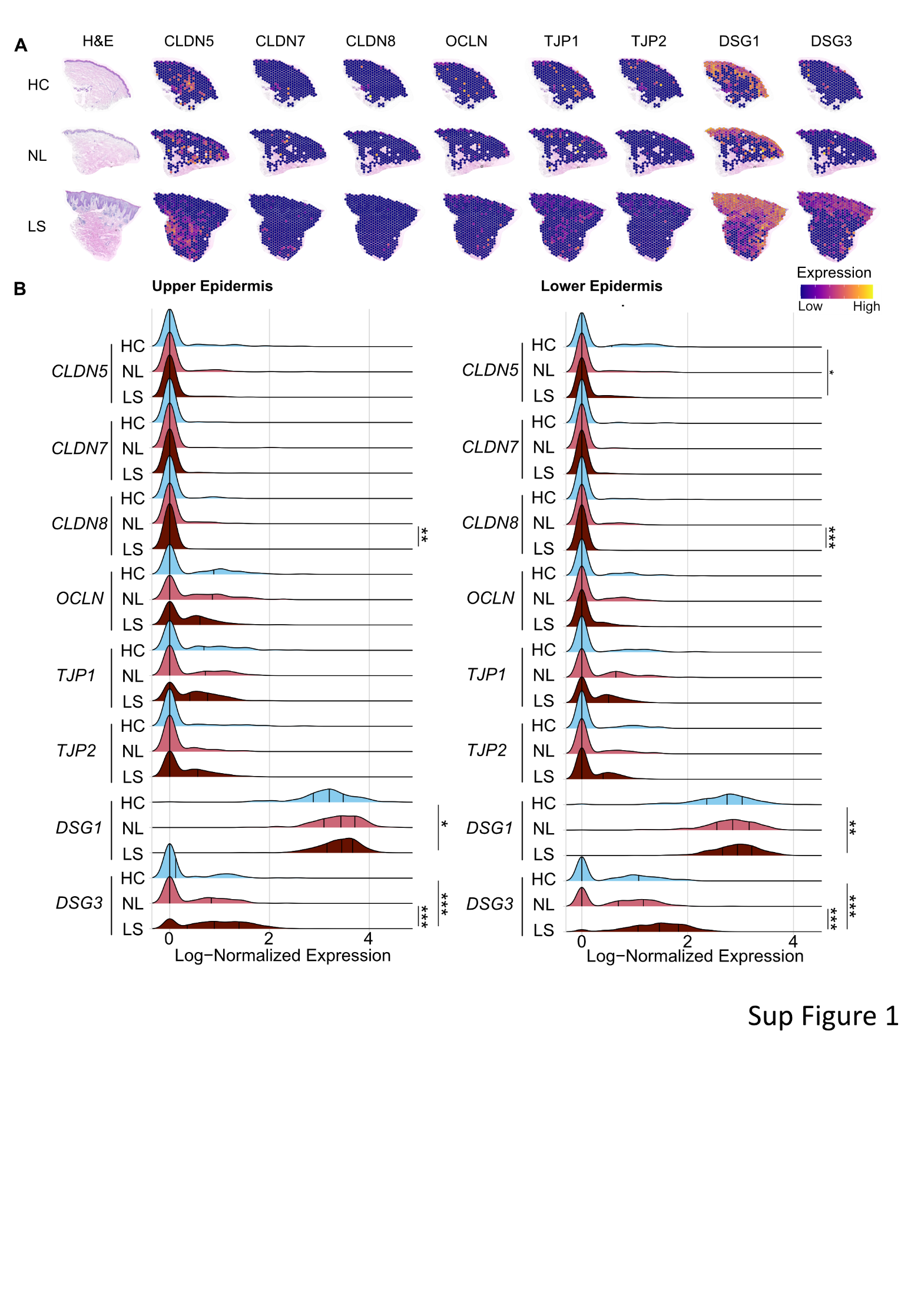


**Figure S1** **Characterization of barrier-related molecules in AD lesional and non-lesional skin**.

(**A)** Spatial feature plots of expression of skin barrier-related genes, claudin-5 (*CLDN5*), claudin-7 (*CLDN7*), claudin-8 (*CLDN8*), occluding (*OCLN*), tight junction protein-1 (*TJP1*), tight junction protein-2 (*TJP2*), desmoglein-1 (*DSG1*), and desmoglein-3 (*DSG3*) in healthy controls (HC), nonlesional AD (NL), and lesional AD (LS). (**B)** Ridgeline plots of the indicated gene expression within the upper and lower epidermis cluster*p ≤ .05, **p ≤ .01, ***p ≤ .001, ****p ≤ .0001, Wilcoxon rank-sum test.


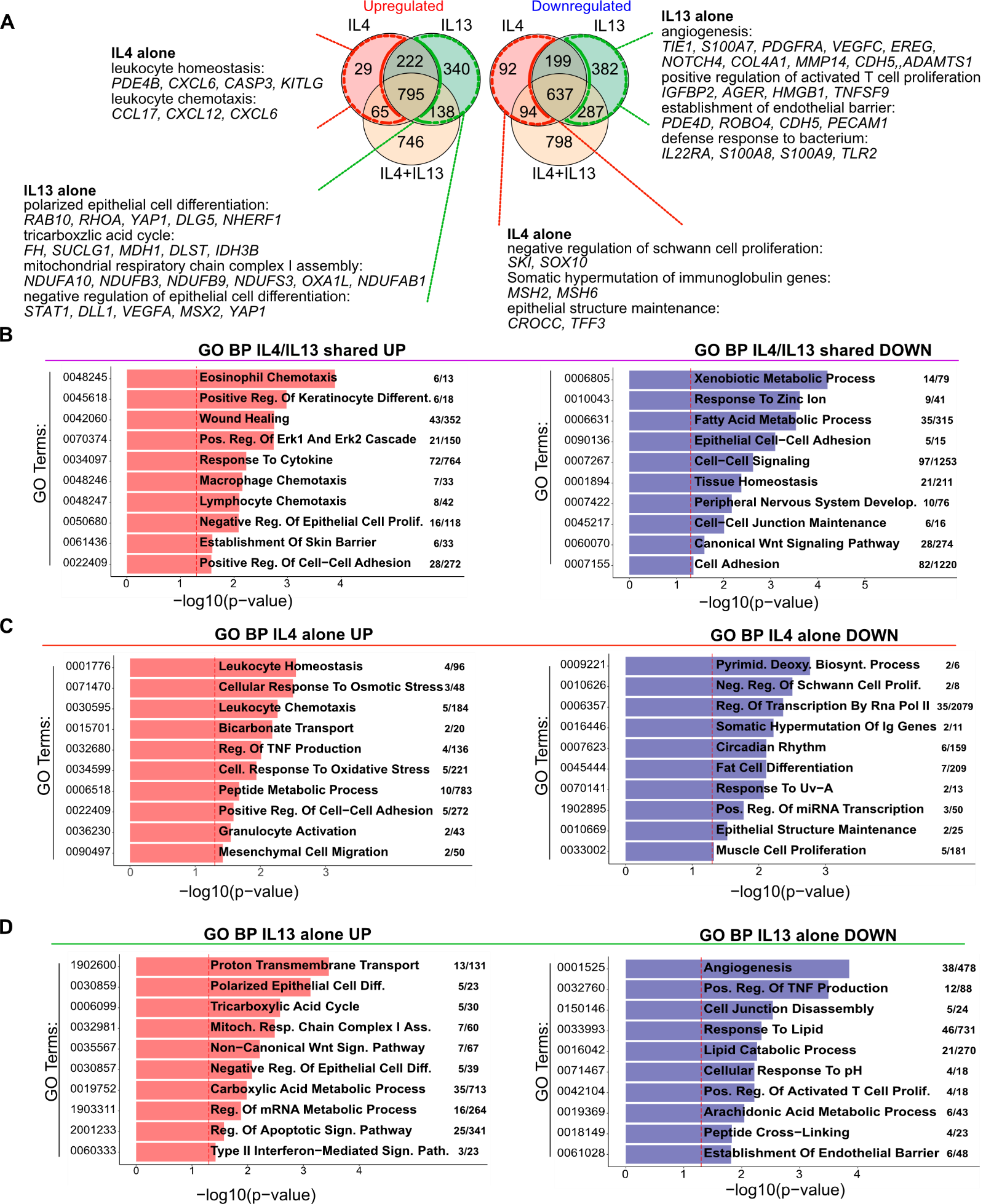


**Figure S2** **Type-2 cytokines induce distinct gene expression profiles in ex-vivo human skin.** (**A)** Venn diagrams illustrate the number of differentially expressed genes (FDR<0.05) in comparison of each stimulation compared to the control. The dotted lines show the differentially expressed genes upon stimulation with IL-4 alone (red) and IL-13 alone (green). The representative genes are indicated. (**B-D)** Representative significant Gene Ontology terms of biological processes (GO BP) associated with the differentially expressed genes (DEGs) shown in the **(B)** IL-4/IL-13 shared; (**C)** IL-4 alone; (**D)** IL-13 alone. The number indicates the number of overlapping differentially expressed genes/number of genes involved in the corresponding biological process.


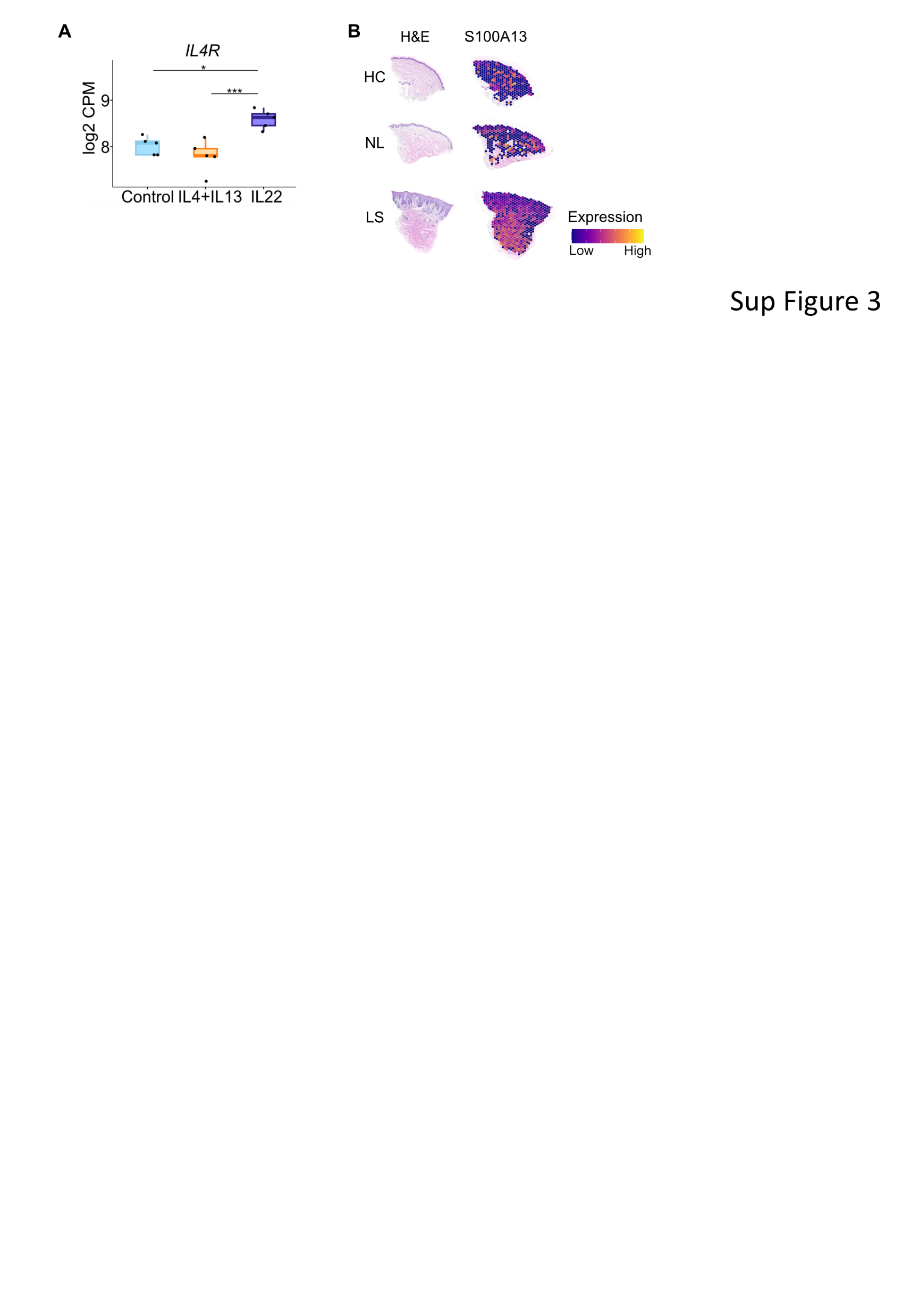


**Figure S3** **IL-22 upregulates IL4RA and antimicrobial peptides**. **(A)** IL4RA expression upon stimulation with IL-4+IL-13, and IL-22, and controls. **(B)** Spatial expression features of S100A13 in healthy controls (HC), non-lesional skin (NL), and lesional skin (LS) of atopic dermatitis.

**
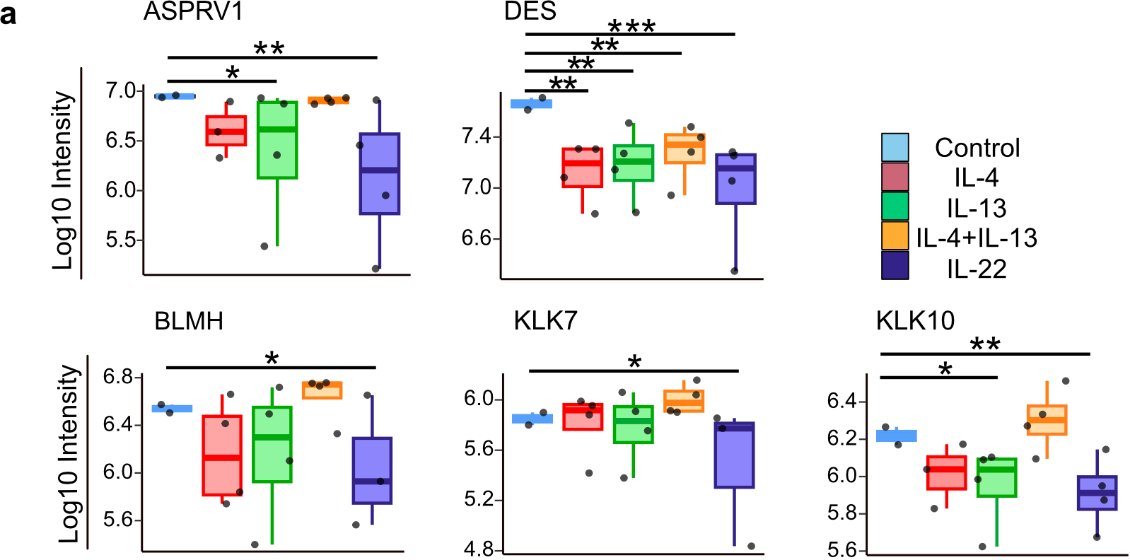
Figure S4** **IL-22 decreases filaggrin-related proteases.** Protein expressions of aspartic peptidase retroviral-like 1 (ASPRV1), desmin (DES), bleomycin hydrolase (BLMH), kallikrein 7 (KLK7), and kallikrein 10 (KLK10) upon stimulation with IL-4 and IL-13 alone, IL-4+IL-13, and IL-22 alone compared to controls. Statistical analysis derived from limma linear model, *p ≤ .05, **p ≤ .01, ***p ≤ .001


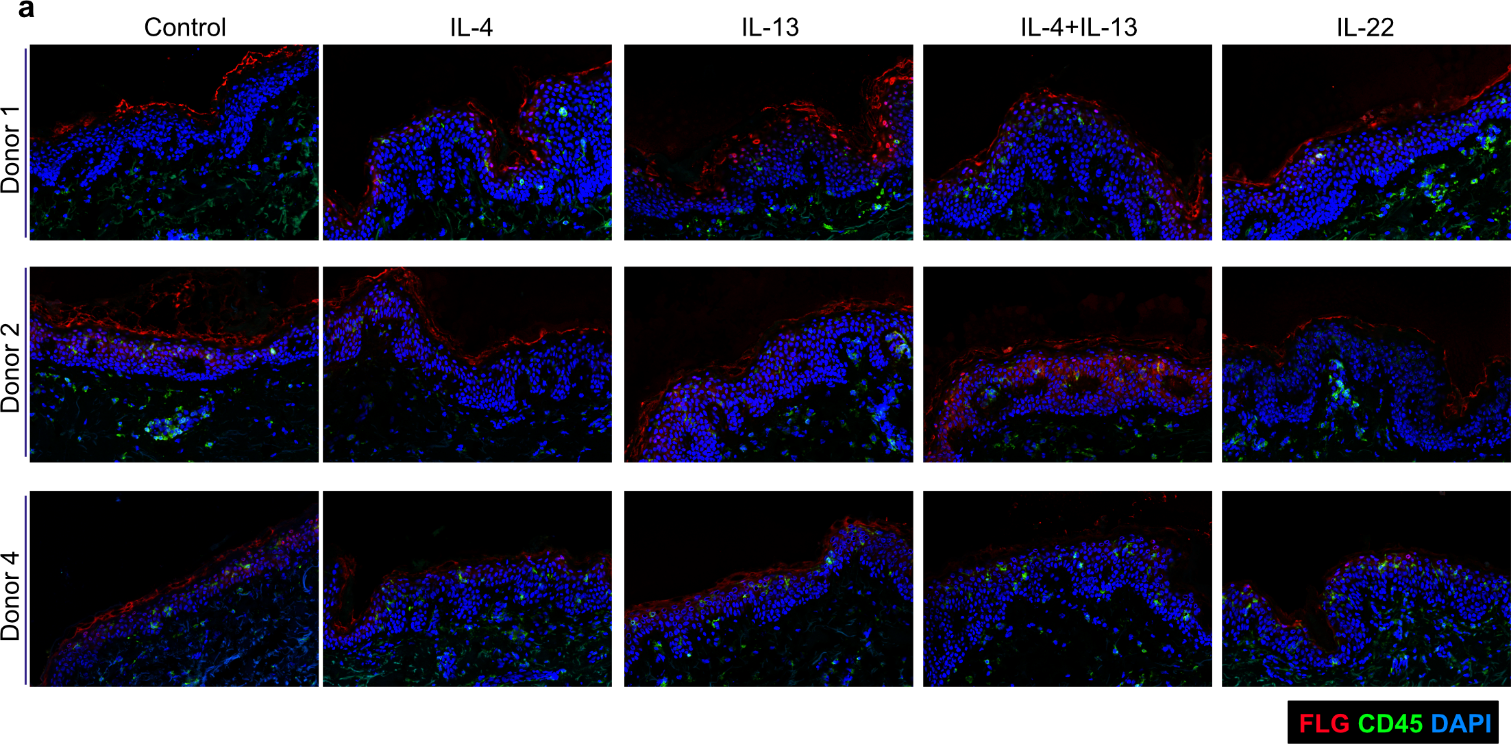


**Figure S5** **IL-4, IL-13, and IL-22 reduced the expression of filaggrin.** Representative immunohistochemistry images of filaggrin expression (red) and CD45 positive cells (green) in the ex-vivo skin treated with the indicated cytokines.
